# 8-Epi-Prostaglandin F2α as a Redox Biomarker in Inflammatory Bowel Disease

**DOI:** 10.3390/biomedicines14051047

**Published:** 2026-05-05

**Authors:** Ioana-Gabriela Dragne, Bogdan Silviu Ungureanu, Dragoș Forțofoiu, Vlad Pădureanu, Lidia Boldeanu, Mohamed-Zakaria Assani, Daniel Cosmin Caragea, Dan Ionuț Gheonea, Venera Cristina Dinescu, Mihail Virgil Boldeanu

**Affiliations:** 1 Doctoral School, University of Medicine and Pharmacy of Craiova, 200349 Craiova, Romania; gabriela.dragne@umfcv.ro (I.-G.D.); dragos.fortofoiu@umfcv.ro (D.F.); mohamed.assani@umfcv.ro (M.-Z.A.); 2 Department of Gastroenterology, University of Medicine and Pharmacy of Craiova, 200349 Craiova, Romania; bogdan.ungureanu@umfcv.ro (B.S.U.); digheonea@gmail.com (D.I.G.); 3 Department of Internal Medicine, University of Medicine and Pharmacy of Craiova, 200349 Craiova, Romania; vlad.padureanu@umfcv.ro; 4 Department of Microbiology, Faculty of Medicine, University of Medicine and Pharmacy of Craiova, 200349 Craiova, Romania; 5 Department of Immunology, Faculty of Medicine, University of Medicine and Pharmacy of Craiova, 200349 Craiova, Romania; mihail.boldeanu@umfcv.ro; 6 Department of Nephrology, Faculty of Medicine, University of Medicine and Pharmacy of Craiova, 200349 Craiova, Romania; daniel.caragea@umfcv.ro; 7 Department of Health Promotion and Occupational Medicine, University of Medicine and Pharmacy of Craiova, 200349 Craiova, Romania

**Keywords:** 8-epi-PGF2α, lipid peroxidation, oxidative stress, GPX1, SOD1, ulcerative colitis, Crohn’s disease, inflammation, antioxidant defense

## Abstract

**Background:** Oxidative stress plays a significant role in inflammatory bowel disease (IBD), yet the clinical relevance of specific lipid peroxidation markers remains insufficiently defined. This study evaluated serum levels of 8-epi-prostaglandin F2α (8-epi-PGF2α), an isoprostane generated through non-enzymatic lipid oxidation, and examined its relationship with antioxidant enzymes and clinical disease activity in ulcerative colitis (UC) and Crohn’s disease (CD). **Methods:** Eighty-seven patients (55 UC and 32 CD) were assessed for serum 8-epi-PGF2α, superoxide dismutase 1 (SOD1), and glutathione peroxidase 1 (GPX1), and classified as having mild, moderate, or severe disease. Statistical analyses included comparative analysis, two-way ANOVA, multiple linear regression, and Ridge logistic regression. To address potential dietary confounding, total energy intake, Mediterranean Diet Score (MDS), and antioxidant supplement use were incorporated into the regression models. **Results:** Serum levels of 8-epi-PGF2α and GPX1 were significantly higher in UC than in CD (*ρ* = 0.001 and *p* = 0.042), and both increased with greater disease severity (*p* < 0.001 and *p* = 0.001). In UC, 8-epi-PGF2α positively correlated with high-sensitivity C-reactive protein (hs-CRP), white blood cells (WBC), and Truelove–Witts Index (TWI), and negatively with hemoglobin (False Discovery Rate (FDR)-adjusted q < 0.100). In CD, it correlated with the Harvey–Bradshaw Index (HBI) and disease duration (FDR-adjusted q < 0.050). Inter-biomarker analyses showed a strong association between 8-epi-PGF2α and GPX1 in UC (*ρ* = 0.677, *p* < 0.0001, FDR < 0.0001), suggesting coordinated activation of oxidative and antioxidant pathways. The observed associations remained consistent after adjustment for dietary factors, supporting the robustness of the findings. Because these results are cross-sectional, they cannot establish causality and should be interpreted with caution. **Conclusions:** Nevertheless, 8-epi-PGF2α emerges as a promising non-invasive biomarker for assessing oxidative stress and disease activity in IBD, with potential clinical applicability for patient monitoring and therapeutic evaluation.

## 1. Introduction

Inflammatory bowel disease (IBD) consists of two primary types: Crohn’s disease (CD) and ulcerative colitis (UC). Both are chronic, relapsing–remitting, immune-mediated inflammatory conditions of the gastrointestinal tract. CD is characterized by transmural inflammation that can affect any part of the GI tract from the mouth to the anus. In contrast, UC is restricted to the colonic mucosa and submucosa, with continuous lesions confined to the colon [1,2].

A key feature of IBD is persistent inflammation caused by dysregulated immune responses targeting luminal antigens. This process involves cytokine signaling, epithelial barrier breakdown, and microbial imbalance. Additionally, oxidative stress worsens tissue damage by harming lipids, proteins, and deoxyribonucleic acid (DNA), thereby sustaining the inflammatory cycle [3,4,5].

IBD is increasingly recognized as a multifactorial disorder involving not only immune dysregulation but also metabolic and mitochondrial alterations that contribute to disease progression. Emerging evidence highlights the role of mitochondrial dysfunction in amplifying oxidative stress, impairing epithelial barrier integrity, and promoting chronic inflammation. In parallel, alterations in the gut microbiota composition and reduced production of protective metabolites, such as short-chain fatty acids, further exacerbate mucosal damage and immune activation. Current therapeutic strategies in IBD include aminosalicylates, corticosteroids, immunomodulators, and biologic agents targeting key inflammatory pathways. Although these treatments have improved disease management, a substantial proportion of patients fail to achieve sustained remission or mucosal healing. Therefore, there is growing interest in identifying novel biomarkers that reflect underlying pathogenic mechanisms beyond classical inflammation, particularly those related to oxidative stress and redox imbalance [6,7].

Reactive oxygen species (ROS) production in inflamed tissues surpasses endogenous antioxidant capacity, especially during chronic inflammation. This oxidative imbalance leads to lipid peroxidation of membrane phospholipids, producing stable secondary products such as F2-isoprostanes. Mechanistically, 8-epi-PGF2α belongs to the F2-isoprostane family and is generated non-enzymatically through free-radical-mediated peroxidation of arachidonic acid esterified in membrane phospholipids. This pathway occurs independently of cyclooxygenase (COX) activity and differs fundamentally from the formation of classical prostaglandins, which are synthesized enzymatically from free arachidonic acid by COX-1 and COX-2. Because of its COX-independent, free-radical-driven origin, 8-epi-PGF2α is considered a highly specific, chemically stable, and reliable in vivo biomarker of oxidative lipid damage [8,9]. Due to their chemical stability and high specificity, F2-isoprostanes, including 8-iso-prostaglandin F2α (8-iso-PGF2α; also known as 8-epi-PGF2α), are considered reliable in vivo biomarkers of oxidative lipid damage. Evidence indicates that lipid peroxidation biomarker levels are higher in patients with IBD than in healthy controls [10].

In IBD patients, measurements in both urine and serum/plasma show that 8-iso-PGF2α is elevated in adults. It is noted that in pediatric cohorts, 8-iso-PGF2α may not differ, but in adult CD and UC, serum/plasma levels are increased [11]. Oxidative stress is now understood not just as a consequence of inflammatory processes but also as a key driver in the development of IBD. It promotes the progression from acute inflammatory episodes to ongoing tissue damage, ultimately leading to fibrosis and stricture formation, particularly in CD. Additionally, oxidative stress influences epithelial regenerative processes. Elevated ROS levels inhibit stem cell growth and impede mucosal repair, which may explain why some patients do not reach deep remission despite receiving optimal anti-inflammatory therapy [12].

Longitudinal studies also indicate that monitoring oxidative stress markers can help in assessing treatment responses. Patients who respond to therapy typically show decreases in lipid peroxidation markers and partial recovery of antioxidant enzyme activity [11]. Oxidative stress may also influence patients’ response to treatment. Those with a higher oxidative burden might experience a less favorable response to immunomodulators and biologics, emphasizing the importance of maintaining redox balance for effective disease management [12,13]. Oxidative stress plays a fundamental role in the initiation and exacerbation of IBD through damaging cellular components and promoting inflammation. Reactive oxygen and nitrogen species released from infiltrating leukocytes contribute significantly to mucosal injury and inflammation within the gastrointestinal tract [1,14,15,16].

In addition to anti-inflammatory and immunosuppressive therapies, several adjunctive strategies have been explored in IBD, including probiotics, postbiotics, and microbiota-targeted interventions. Certain probiotic strains may improve epithelial barrier integrity, modulate mucosal immune responses, and reduce oxidative stress, while postbiotic metabolites have been shown to exert anti-inflammatory and antioxidant effects in experimental models. Although their efficacy varies across studies, these adjuvants are increasingly considered supportive options within a multimodal therapeutic approach to IBD [17,18,19,20,21].

Antioxidant enzymes like superoxide dismutase (SOD) and glutathione peroxidase (GPX) are crucial components of the body’s natural defense against oxidative stress. In UC, evidence shows increased ROS levels and decreased antioxidant capacity in mucosal tissue, including reduced SOD activity. The impact of oxidative stress and the status of antioxidant defenses in UC are demonstrated through changes in enzyme activities, particularly SOD, catalase, and glutathione peroxidase, which may influence disease progression [22,23].

Given the limited and often diverse data on 8-iso-PGF2α in IBD cohorts, this study aims to measure serum levels of 8-iso-PGF2α, GPX1, and SOD1 in patients with CD and UC. The analysis assesses whether 8-iso-PGF2α offers additional discriminative power beyond traditional inflammatory markers in differentiating between CD and UC. Additionally, correlations between oxidative stress markers and clinical phenotypes are explored to evaluate the potential of 8-iso-PGF2α as a biomarker for distinguishing these conditions.

## 2. Materials and Methods

### 2.1. Study Design

This study was carried out following approval from the Committee of Ethics and Academic and Scientific Deontology at the University of Medicine and Pharmacy in Craiova, number 236/20 June 2024. Each patient signed an informed consent form to participate in our study. All procedures complied with the ethical standards of the responsible institutional committees for human research and the Helsinki Declaration of 1975–2013, as revised in 2008.

### 2.2. Patient Selection

This retrospective study was conducted at the Gastroenterology Clinic of the Emergency County Clinical Hospital in Craiova, between October 2024 and March 2025.

We included eighty-seven patients with IBD (55 with UC and 32 with CD) aged 19–77 years in the study. When they consulted a specialist (clinic or hospital), the patients underwent clinical examination, lower gastrointestinal endoscopy, and histopathological examination of biopsy samples. Biological samples were collected from the patients and included in the study.

Groups were organized based on specific criteria. The study included patients aged 18 or older with active UC or CD. It excluded participants with hematologic, metabolic, infectious, neoplastic, other organic (e.g., heart disease, lung disease, hepatosplenic disease, renal insufficiency), or autoimmune diseases.

A structured form was created for each UC or CD patient, including contact info, demographics, medical history, symptoms, lab results, phenotypic class, complications, disease duration, surgical history, medication, and current treatment.

The UC or CD patients received 5-aminosalicylates (5-ASA), a glucocorticoid (budesonide), and steroid medications (methylprednisolone, prednisone), as well as immunomodulatory therapies, including thiopurines, methotrexate, azathioprine, cyclosporine, and tacrolimus.

Patients with UC were categorized according to the Montreal system [24,25], which classifies the disease extent endoscopically: E1—proctitis; E2—left colitis; E3—extensive colitis (see Table 1). To assess disease activity, we used the Truelove and Witts severity index (TWI):

- TWI score ≤ 5, corresponding to a Mild disease activity (Bowel movements/number per day; therefore, less than 4; Blood in stools—No more than small amounts of blood; Pyrexia (temperature higher than 37.8 °C)—No; Pulse rate higher than 90 bpm—No; Anemia—No; ESR (mm/hour)—30 or below):
- TWI score = 5–9, corresponding to a Moderate disease activity (Bowel movements/number per day)—4–6; Blood in stools—Between mild and severe; Pyrexia (temperature greater than 37.8 °C)—No; Pulse rate greater than 90 bpm—No; Anemia—No; ESR (mm/hour)—30 or below);
- TWI score ≥ 9, corresponding to a Severe disease activity (Bowel movements/number per day*)*—6 or more along with a minimum of 1 systemic upset aspect (marked with * below): Blood in stools—Visible blood; Pyrexia (temperature greater than 37.8 °C)—Yes; Pulse rate greater than 90 bpm—Yes; Anemia—Yes; ESR (mm/hour)—Above 30) [26,27].

**Table 1:** Montreal classification of the ulcerative colitis (UC) and Crohn’s disease (CD) patients.

**Parameters**	**Ulcerative Colitis (*n* = 55), *n* (%)**
E1—Ulcerative proctitis	15 (32.61%)
E2—Left-sided UC (distal UC)	18 (39.13%)
E3—Extensive UC (pancolitis)	13 (28.26%)
	**Crohn’s disease (*n* = 32), *n* (%)**
*Age at diagnosis*	
A1 below 16 years	-
A2 between 17 and 40 years	16 (50.00%)
A3 above 40 years	16 (50.00%)
*Location*	
L1 ileal	9 (28.12%)
L2 colonic	11 (34.38%)
L3 ileocolonic	12 (37.50%)
*Behavior*	
B1 non-stricturing, non-penetrating	13 (40.63%)
B2 stricturing	9 (28.12%)
B3 penetrating	10 (31.25%)

Each patient with CD was categorized based on the Montreal classification (Table 1) [28]:

- age at diagnosis (A1: <16 years, A2: 17–40 years, A3: >40 years),
- location of the lesions (L1: ileal, L2: colonic, L3: ileo-colonic, L4: upper digestive tract, p: perianal),
- pattern (type) of the lesions (B1: non-stenosing, non-penetrating, B2: stenosing, B3: penetrating).

For each case of CD, the Harvey–Bradshaw index (HBI) [24] was calculated, which is useful for assessing disease activity and flare severity (Table 2):

- general condition (0 = good, 1 = slightly altered, 2 = very altered, 3 = severe);
- abdominal pain (0 = absent, 1 = mild, 2 = moderate, 3 = severe);
- number of liquid stools per day;
- presence of abdominal masses (0 = absent, 1 = possible, 2 = delimited, 3 = plaque);
- complications (1 × arthritis, uveitis, erythema nodosum, oral ulcers, pyoderma gangrenosum, fissures, fistulas, anal abscesses).

**Figure 1:**
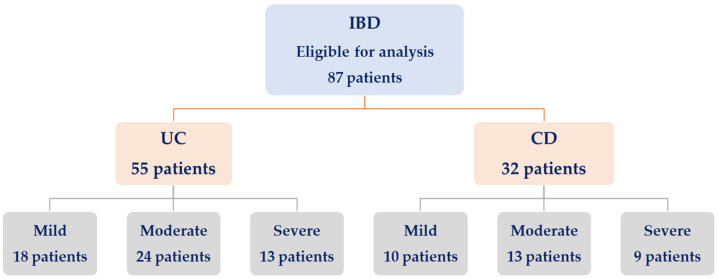
Flow diagram of participant distribution across disease severity groups (mild/moderate/severe).

Depending on the score achieved, each patient was categorized into the respective activity type, as follows (refer to Table 2, Figure 1):

- HBI score 5–7, corresponding to Mild disease activity
- HBI score 8–16, corresponding to Moderate disease activity
- HBI score > 16, corresponding to Severe disease activity.

**Table 2:** Assessing the disease activity of the UC and CD patients.

Parameters	UC (*n* = 55)	CD (*n* = 32)
Truelove and Witts index (TWI), *n* (mean ± stdev)		
TWI score ≤ 5—mild activity	3.17 ± 0.62	-
TWI score 5–9—moderate activity	7.33 ± 1.17	-
TWI score > 9—severe activity	11.92 ± 1.50	-
Harvey–Bradshaw index (HBI), *n* (mean ± stdev)		
HBI score 5–7 —mild activity	-	6.00 ± 0.82
HBI score 8–16—moderate activity	-	11.62 ± 2.57
HBI score > 16—severe activity	-	18.11 ± 0.93

### 2.3. Sample Collection

The biological samples obtained from patients included about 5 mL of venous blood collected into additive-free tubes (Becton, Dickinson Vacutainer, Franklin Lakes, NJ, USA). Centrifugation (Hermle AG, Gosheim, Baden-Württemberg, Germany) at 3000× *g* for 10 min within 4 h of collection separated the clot, following the standard technique. Patients’ serum sample tubes were labeled, sealed, and stored at −20 °C to −80 °C for long-term processing. Prior to processing, frozen specimens were thawed at ambient temperature and subjected to a single freeze–thaw cycle.

For a complete blood count (CBC), peripheral venous blood was taken into ethylenediaminetetraacetic acid (EDTA) vacutainer tubes to measure white blood cells (neutrophils, lymphocytes, monocytes), red blood cells, platelets, hemoglobin, and hematocrit.

### 2.4. Immunological Assessment

To quantify serum levels of 8-epi-PGF2α, SOD1, and GPX1, the Enzyme-Linked Immunosorbent Assay (ELISA) was performed in the Immunology Laboratory of the University of Medicine and Pharmacy of Craiova.

We used commercially available test kits from Elabscience (Houston, TX, USA), designed for each of the following mediators:

- 8-epi-PGF2α (8-Epi-Prostaglandin F2 Alpha) ELISA Kit (Catalog No.: E-EL-0041; Product Link: https://www.elabscience.com/p/8-epi-pgf2-8-epi-prostaglandin-f2-alpha-elisa-kit--e-el-0041 (accessed on 5 April 2026); Sensitivity: 9.38 pg/mL; Detection range: 15.63–1000 pg/mL; Specificity: No significant cross-reactivity or interference between 8 epi-PGF2α and analogs was observed; Intra-/Inter-Assay CV (%): Coefficient of variation is <10%; https://789.bio/ea/OC4Om5) (accessed on 5 April 2026);
- Human GPX1 (Glutathione Peroxidase 1) ELISA Kit (Catalog No.: E-EL-H5410; Product Link: https://www.elabscience.com/p/human-gpx1-glutathione-peroxidase-1-elisa-kit--e-el-h5410 (accessed on 5 April 2026); Sensitivity: 18.75 pg/mL; Detection range: 31.25–2000 pg/mL; Specificity: No significant cross-reactivity or interference between Human GPX1 and analogs was observed; Intra-/Inter-Assay CV (%): Coefficient of variation is <10%; https://789.bio/ea/ybzzf1) (accessed on 5 April 2026);
- Human SOD1 (Superoxide Dismutase 1, Soluble) ELISA Kit (Catalog No.: E-EL-H1113; Product Link: https://www.elabscience.com/p/human-sod1-superoxide-dismutase-1-soluble-elisa-kit--e-el-h1113 (accessed on 5 April 2026); Sensitivity: 37.5 pg/mL; Detection range: 62.5–4000 pg/mL; Specificity: No significant cross-reactivity or interference between Human SOD1 and analogs was observed; Intra-/Inter-Assay CV (%): Coefficient of variation is <10%; https://789.bio/ea/vnXzfH) (accessed on 5 April 2026).

Following thawing, each sample was diluted as per the manufacturer’s guidelines and the recommended procedure, using a standard optical analyzer set to 450 nm.

### 2.5. Complete Blood Counting (CBC)

An automatic hematology analyzer (Alinity, Abbott, Abbott Park, IL, USA) used flow cytometry to differentiate and count five white blood cell (WBC) types, neutrophils (NEU), monocytes (MON), lymphocytes (LYM), basophils, providing an extended leukocyte formula, and platelets (PLT).

ESR was measured using the Westergren method (ESR tubes, Becton Dickinson, Franklin Lakes, NJ, USA). High-sensitivity CRP (hs-CRP) was determined with an automated chemiluminescent immunoassay analyzer (Cobas e411, Roche Diagnostics GmbH, Mannheim, Germany).

### 2.6. Dietary Assessment

Dietary intake was retrospectively assessed using a food frequency questionnaire (FFQ) adapted from the European Prospective Investigation into Cancer and Nutrition (EPIC) study, covering habitual intake over the four weeks preceding blood sampling. The questionnaire included major food groups relevant to assessing dietary patterns, such as vegetables, fruits, legumes, cereals, fish, meat, dairy products, alcohol, and olive oil consumption. Daily intake of each food group was estimated in grams per day based on standard portion sizes and reported consumption frequency. Total energy intake (kcal/day) was calculated using standard food composition tables [29,30,31].

A Mediterranean Diet Score (MDS, range 0–9) was computed as a continuous variable based on cohort-specific median intake values. For beneficial food groups (vegetables, fruits, legumes, cereals, fish), participants received 1 point for intake at or above the cohort median. For detrimental components (meat and dairy), 1 point was assigned for intake below the median. Moderate alcohol consumption was scored according to predefined thresholds, and use of olive oil as the main added fat was used as a proxy for the lipid component. In addition, the use of antioxidant supplements (yes/no) was recorded and included as a separate variable [32,33,34].

To account for potential dietary confounding, total energy intake, MDS, and antioxidant supplement use were incorporated into all multivariable regression and predictive models [35,36,37]. Although dietary intake was assessed retrospectively, this approach allowed adjustment for major dietary patterns that could influence oxidative stress biomarkers.

### 2.7. Statistical Analysis

Patient data management and processing were carried out using Microsoft Excel and its Data Analysis module, while statistical analysis was performed with GraphPad Prism 11.0.0 (84) (LLC, San Diego, CA, USA).

We evaluated data normality using the D’Agostino–Pearson omnibus normality test. For normally distributed variables, we report the mean with its standard deviation (SD), while categorical variables are presented as percentages.

#### 2.7.1. Pairwise Comparisons

After the two-way ANOVA revealed significant main effects, nonparametric pairwise comparisons (Mann–Whitney U test) were performed separately for UC and CD between the mild, moderate, and severe activity groups. The analysis focused on the two markers showing significant main effects in the ANOVA—8-epi-PGF2α and GPX1—to identify which activity categories differed significantly in median values. To reduce the risk of false positives from multiple testing, *p*-values were adjusted using the Benjamini–Hochberg false discovery rate (FDR) correction within each marker-disease pair. Results are presented as adjusted *p*-values, significance levels (ns, *p* < 0.05, *p* < 0.01, *p* < 0.001, *p* < 0.0001), and the direction of change (↑ for higher values in the more active group). Only comparisons with adjusted *p* < 0.05 were deemed statistically significant.

#### 2.7.2. Regression Analyses

Multiple linear regression (MLR) analysis was performed to identify independent predictors of serum oxidative stress markers (8-epi-PGF2α, SOD1, and GPX1) in patients with UC and CD. Separate regression models were constructed for each disease group and biomarker to account for disease-specific patterns. Each oxidative stress marker (M) was used as the dependent variable, while clinical, biochemical, and pre-analytical parameters were included as independent variables according to the following model:

M ~ Status(UC/CD) + Age + Sex + Smoking + Body mass index (BMI) + Disease duration + hs-CRP + Albumin (ALB) + Energy intake (kcal/day) + MDS continuous + Antioxidant supplement use (0/1).

Categorical variables (sex, smoking, supplement use) were dummy-coded before inclusion in the model. Continuous variables were tested for normality and, if necessary, log-transformed before regression. All models were fitted using the ordinary least squares (OLS) method with HC3 robust standard errors to correct for potential heteroscedasticity.

Separate models were generated for UC and CD to examine disease-specific associations. For each model, the regression coefficient (*β*), 95% confidence interval (CI), and *p*-value were calculated. Model adequacy was assessed using the coefficient of determination (R^2^) and the adjusted R^2^. Statistical significance was set at *p* < 0.05. Predictors with *p* < 0.05 were interpreted as independent factors associated with oxidative stress or antioxidant activity. No variable selection or stepwise elimination was applied, as all predictors were entered simultaneously to evaluate their relative contributions within the predefined biological model.

While the multiple linear regression models identified disease-specific determinants of oxidative and antioxidant activity, we subsequently assessed whether these biomarkers were independently associated with clinical disease activity. To evaluate the independent contribution of oxidative and antioxidant biomarkers to disease severity, separate binary Ridge logistic regression models (severe vs. moderate/mild) were constructed for UC and CD. All biochemical, clinical, and pre-analytical parameters, including 8-epi-PGF2α, SOD1, GPX1, age, sex, smoking, BMI, disease duration, hs-CRP, ALB, treatment variables (5-ASA, steroids, immunomodulators, biologics, or NSAIDs), as well as total energy intake, MDS, and antioxidant supplement use, were entered as covariates. Both models were standardized and regularized using an L2 (Ridge) penalty to minimize overfitting and to account for multicollinearity among predictors. Standardized coefficients (*β*) and the corresponding odds ratios per 1 SD (OR per 1 SD) were derived to quantify the independent influence of each biomarker and covariate on severe disease activity. A positive *β* (OR > 1) indicates increased odds of severe disease, whereas a negative *β* (OR < 1) denotes a protective or inverse association. Model performance was further evaluated using receiver operating characteristic (ROC) analysis, and the area under the curve (AUC) and Youden’s index were calculated to assess diagnostic accuracy, sensitivity, and specificity.

#### 2.7.3. Correlation Analysis

Spearman’s rank correlation analysis was conducted separately for UC and CD to evaluate associations between oxidative stress biomarkers (8-epi-PGF2α, SOD1, GPX1) and clinical or biochemical variables (age, BMI, disease duration, hs-CRP, ALB). The nonparametric Spearman method was selected because the data were nonnormal. Categorical variables such as sex and smoking were excluded. To correct for multiple testing, *p*-values were adjusted using the Benjamini–Hochberg procedure to control the FDR, applied independently for UC and CD. Correlations with q ≤ 0.05 were considered significant, and those with q ≤ 0.10 were considered trends. Results were expressed as Spearman’s *ρ* with corresponding *p*- and q-values and visualized as heatmaps.

## 3. Results

### 3.1. Clinical and Demographic Characteristics of Patients with UC and CD

Patients with UC and CD were comparable in age (47.0 ± 17.5 years vs. 42.4 ± 13.4 years, *p* = 0.2955), sex distribution (*p* = 0.057), and area of residence (*p* = 0.863) (Table 3). Smoking and alcohol consumption did not differ significantly across the three groups (*p* = 0.512 and *p* = 0.449, respectively). However, when comparing UC and CD subgroups, the proportion of smokers was higher in CD (62.5%) than in UC (38.2%, *p* = 0.028). The median disease duration was significantly longer in UC (median 13 years [1,2,3,4,5,6,7,8,9,10,11,12,13,14,15,16,17,18,19,20,21,22]) than in CD (7.5 years [2,3,4,5,6,7,8,9,10,11,12,13,14,15], *p* < 0.0001), indicating a more chronic course in UC. BMI differed significantly across groups (*p* < 0.0001), with both UC and CD patients showing higher values compared to controls, while no significant difference was observed between UC and CD (*p* = 0.345).

Compared to controls, both UC and CD patients exhibited significantly elevated WBC and NEU counts (*p* < 0.0001), indicating systemic inflammation. No significant differences were observed between UC and CD for these parameters (*p* > 0.05). However, CD patients had significantly higher LYM (*p* = 0.029) and MON (*p* = 0.001) counts.

Hemoglobin (Hb) levels differed significantly across groups (*p* = 0.0001), with lower values observed in both UC and CD compared to controls, consistent with anemia associated with chronic inflammation. Red cell distribution width (RDW) was significantly higher in UC (15.0 ± 2.6 vs. 13.1 ± 1.5, *p* < 0.0001), indicating greater anisocytosis due to chronic inflammation. Mean corpuscular hemoglobin (MCH) was lower in CD (*p* = 0.003). Mean corpuscular hemoglobin concentration (MCHC) did not differ significantly between groups (*p* = 0.282).

PLT count did not differ significantly across groups (*p* = 0.052). However, platelet distribution width (PDW) and mean platelet volume (MPV) showed significant differences (*p* = 0.0005 and *p* = 0.034, respectively), with CD patients exhibiting higher PDW and lower MPV values than UC patients.

Serum ALB levels were significantly lower in both UC and CD compared to controls (*p* < 0.0001), reflecting impaired nutritional status. Inflammatory markers, including hs-CRP, ESR, and fibrinogen, were significantly elevated across groups (*p* < 0.001), confirming a strong systemic inflammatory response in IBD. Differences between UC and CD for these markers were less pronounced and should be interpreted cautiously.

### 3.2. Comparing the Oxidative Stress Marker Values Between the UC, CD, and Control Groups

**Figure 2:**
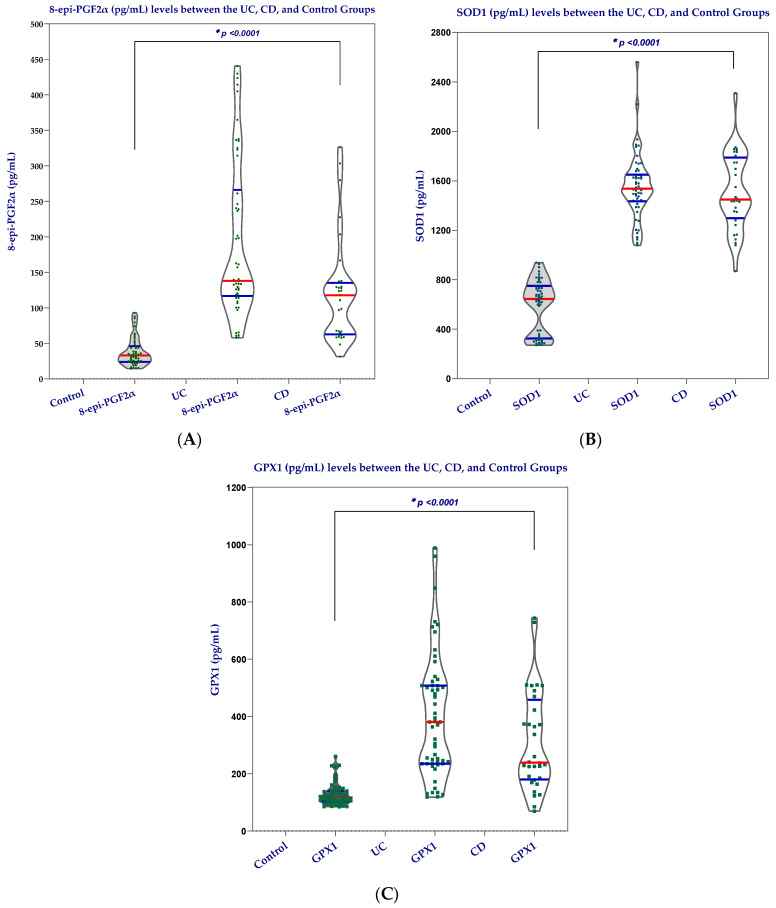
Comparing the oxidative stress marker values between the ulcerative colitis (UC), Crohn’s disease (CD), and control groups: (**A**) 8-epi-prostaglandin F2α (8-epi-PGF2α) (pg/mL), (**B**) superoxide dismutase 1 (SOD1) (pg/mL), (**C**) glutathione peroxidase 1 (GPX1) (pg/mL). The violin plot illustrates the distribution of these biomarkers, with horizontal red lines indicating the median values and horizontal blue lines representing the quartiles.

Oxidative stress and antioxidant biomarkers differed significantly across the three groups (Table 4 and Figure 2). Serum levels of 8-epi-PGF2α were markedly elevated in both UC and CD compared to controls (median 137.95 and 117.84 pg/mL vs. 32.97 pg/mL, *p* < 0.0001), indicating increased lipid peroxidation in IBD. Although UC patients showed higher values than CD, both disease groups demonstrated a substantial increase relative to controls.

Similarly, SOD1 levels were significantly higher in UC and CD than in controls (1537.40 and 1447.50 pg/mL vs. 645.02 pg/mL, *p* < 0.0001), reflecting activation of the enzymatic antioxidant defense system in response to oxidative stress.

GPX1 concentrations followed the same pattern, with markedly higher values in UC and CD compared to controls (380.57 and 239.25 pg/mL vs. 119.68 pg/mL, *p* < 0.0001). The increase was more pronounced in UC, suggesting a stronger compensatory antioxidant response in this subgroup.

Overall, these findings indicate a consistent pattern of increased oxidative stress and activation of antioxidant mechanisms in IBD patients compared to healthy controls, with UC showing a more pronounced redox imbalance.

#### 3.2.1. Oxidative Stress Markers in Relation to Disease Activity

A two-way ANOVA was used to evaluate the effects of marker type (row factor: 8-epi-PGF2α, SOD1, and GPX1) and disease activity (column factor: mild, moderate, or severe) on oxidative stress profiles in patients with UC and CD.

In patients with UC (Table 5), serum levels of 8-epi-PGF2α, SOD1, and GPX1 increased progressively with disease severity. In UC, the row factor representing differences among markers (8-epi-PGF2α, SOD1, GPX1) did not reach statistical significance (*p* > 0.05), indicating that overall, the three oxidative stress markers showed similar variability within the group.

However, the column factor—reflecting the effect of disease activity—was statistically significant for 8-epi-PGF2α (F (2, 29) = 0.027, *p* < 0.0001) and GPX1 (F (2, 29) = 8.573, *p* = 0.001), but not for SOD1 (F (2, 29) = 0.894, *p* = 0.420). These results show that disease activity significantly affects the levels of 8-epi-PGF2α and GPX1, which both increase progressively from mild to severe UC, while SOD1 activity remains relatively stable.

The observed increase in 8-epi-PGF2α highlights enhanced lipid peroxidation, and the parallel rise in GPX1 suggests a compensatory antioxidant response associated with inflammatory activity.

In CD, the same pattern was observed. The row factor was not significant for any marker, indicating that inter-marker differences were not statistically significant.

In contrast, the column factor was significant for 8-epi-PGF2α (F (2, 17) = 12.34, *p* = 0.001) and GPX1 (F (2, 17) = 22.13, *p* < 0.0001), confirming that disease activity strongly influences these markers, which increase with inflammatory severity. Meanwhile, SOD1 remained unchanged (F (2, 17) = 0.554, *p* = 0.585).

Therefore, in both UC and CD, the oxidative stress marker 8-epi-PGF2α and the antioxidant enzyme GPX1 show a clear upward trend with increasing disease activity, while SOD1 does not exhibit significant changes.

Taken together, these findings show that disease activity (column factor) exerts a greater influence on oxidative stress than marker type, highlighting the simultaneous activation of oxidative and antioxidant pathways during disease flares.

**Figure 3:**
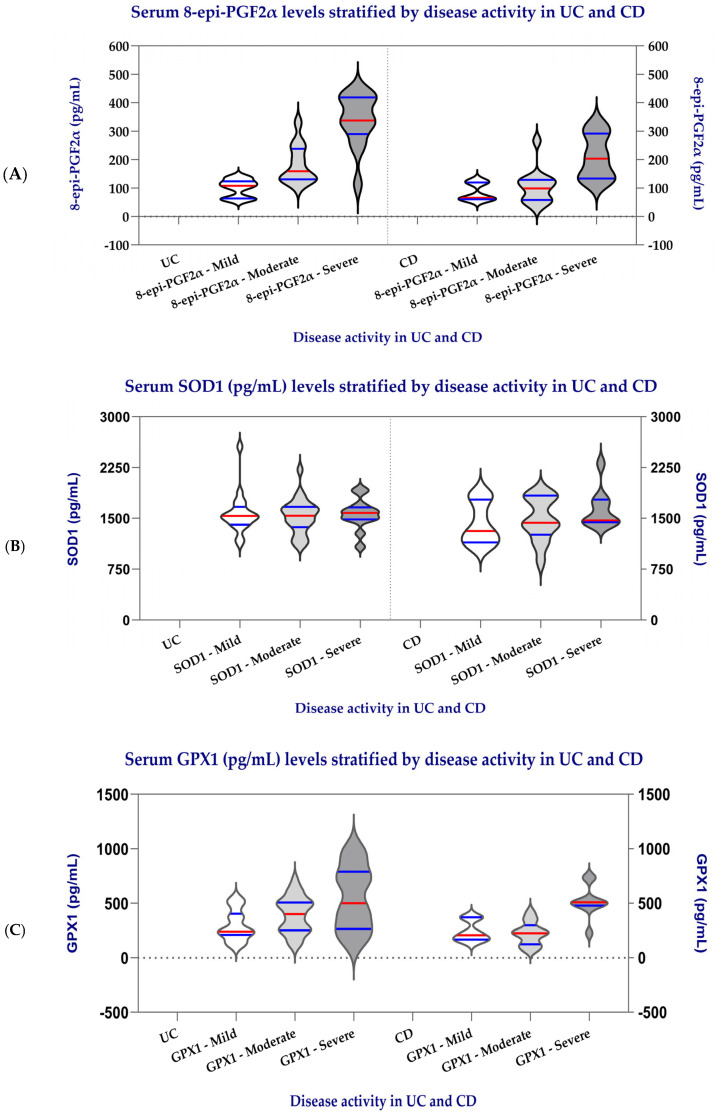
Comparing the oxidative stress marker values of serum 8-epi-PGF2α pg/mL (**A**), SOD1 pg/mL (**B**), and GPX1 pg/mL (**C**) levels stratified by disease activity in ulcerative colitis (left) and Crohn’s disease (right). Each violin plot represents an individual patient. The violin plot illustrates the distribution of these biomarkers, with horizontal red lines indicating the median values and horizontal blue lines representing the quartiles.

To visually illustrate the distribution of oxidative stress markers across disease activity categories, scatter dot plots were generated for 8-epi-PGF2α, GPX1, and SOD1 in UC and CD (Figure 3). In both diseases, 8-epi-PGF2α and GPX1 showed a clear stepwise increase from mild to moderate and severe activity, with higher dispersion and higher median values in the severe group, consistent with the results of the comparative analyses and trend tests. By contrast, SOD1 displayed less pronounced differences across activity categories, with overlapping distributions between mild and moderate disease, particularly in CD.

##### Post Hoc Analysis

Given the significant results from the two-way ANOVA test in patients with UC and CD, we performed post hoc pairwise comparisons (Sidak-adjusted two-way ANOVA test, using the Benjamini–Hochberg false discovery rate (FDR) correction within each marker × disease combination) for 8-epi-PGF2α and GPX1, separately for UC and CD, comparing mild vs. moderate, mild vs. severe, and moderate vs. severe (Table 6).

Post hoc multiple comparisons (Sidak-adjusted two-way ANOVA) revealed a significant increasing trend in 8-epi-PGF2α levels across disease activity stages in UC, with all pairwise comparisons statistically significant (*p* < 0.0001). In CD, 8-epi-PGF2α levels were notably higher in severe disease than in mild (*p* = 0.002) and moderate (*p* = 0.002) disease, indicating increased lipid peroxidation linked to inflammation.

GPX1 activity showed no statistically significant differences across UC activity levels, though a slight upward trend was observed in severe disease (*p* = 0.051). Conversely, in CD, GPX1 activity was significantly higher in severe cases than in mild (*p* = 0.001) or moderate (*p* = 0.001) cases, indicating an adaptive antioxidant response to oxidative stress.

#### 3.2.2. Oxidative Stress Markers Related to Disease Extent and Pattern

A two-way ANOVA was used to assess the effects of marker type (row factor) and disease extent or behavior (column factor) on oxidative stress parameters.

##### Crohn’s Disease—Extent of Disease (L1–L3)

Row factor (marker type)—the row factor was not statistically significant (*p* > 0.05), indicating that the three oxidative markers (8-epi-PGF2α, SOD1, GPX1) exhibited comparable overall variability across disease-extent categories (Table 7). This suggests that oxidative stress and antioxidant responses fluctuate in parallel across the different molecular systems evaluated.

Column factor (disease extent L1–L3)—the column factor showed no significant effect (*p* = 0.128 for 8-epi-PGF2α, *p* = 0.470 for SOD1, *p* = 0.260 for GPX1). However, higher mean levels of 8-epi-PGF2α and GPX1 were observed in colonic and ileocolonic forms (L2, L3) compared to the ileal phenotype (L1). This trend suggests that colonic involvement may increase lipid peroxidation and trigger compensatory antioxidant activation, likely due to greater bacterial-driven oxidative stress and luminal exposure to ROS.

##### Crohn’s Disease—Behavior (B1–B3)

Row factor (marker type)—the row factor remained non-significant, confirming that none of the three oxidative markers differ overall in their expression patterns across behavioral phenotypes (Table 7). This indicates that both pro-oxidant and antioxidant systems change together, with neither being dominant.

Column factor was also not significant (all *p* > 0.05), but median 8-epi-PGF2α values tended to increase in stricturing (B2) and penetrating (B3) subtypes. At the same time, SOD1 and GPX1 decreased slightly in these advanced forms, suggesting depletion of antioxidants in the context of chronic transmural inflammation and fibrotic remodeling. This non-significant trend indicates biological, rather than statistical, differentiation—probably due to small sample sizes within subgroups.

##### Ulcerative Colitis—Extent of Disease (E1–E3)

Row factor (marker type)—the row factor was not significant, meaning that 8-epi-PGF2α, SOD1, and GPX1 varied in a coordinated manner across extent categories. This coherence supports the notion of a linked redox network, in which both pro-oxidant and antioxidant components respond proportionally to the inflammatory burden.

Column factor—the column factor reached statistical significance (*p* = 0.006 for 8-epi-PGF2α), demonstrating a strong effect of disease extent on oxidative stress levels. 8-epi-PGF2α concentrations rose steadily from proctitis (E1) to left-sided (E2) and extensive (E3) colitis, confirming that lipid peroxidation intensity parallels mucosal involvement (Table 7). This indicates a progressive but significant increase, only when moving from limited forms (proctitis, left colitis) to extensive colitis (E3).

SOD1 and GPX1 showed mild, non-significant upward trends (*p* = 0.162 and 0.729), suggesting a compensatory antioxidant response that may lag behind the pro-oxidant stimulus.

##### Post Hoc Analysis

Given the significant results from the two-way ANOVA test in patients with UC, we performed post hoc pairwise comparisons (Sidak-adjusted two-way ANOVA test, using the Benjamini–Hochberg (BH) with FDR correction) for 8-epi-PGF2α comparing mild vs. moderate, mild vs. severe, and moderate vs. severe (Table 8).

In UC, pairwise post hoc testing (Sidak with BH correction) showed that 8-epi-PGF2α levels were higher in E3 than in E1 and E2 (adjusted *p* < 0.05), whereas E1 versus E2 was not significant. Median values increased progressively from E1 to E3. 8-epi-PGF2α increases gradually with the extent of inflammation, but the differences become clearly significant only when the damage extends to a large part of the colon.

### 3.3. Regression Analyses

#### 3.3.1. Multiple Linear Regression

To further explore the independent determinants of oxidative stress biomarkers, MLR analyses were performed separately for UC and CD. Each biomarker (8-epi-PGF2α, SOD1, and GPX1) was entered as a dependent variable, while demographic, clinical, inflammatory, and dietary variables were included as independent predictors. In addition to age, sex, smoking status, BMI, disease duration, hs-CRP, and ALB, the revised models also included total energy intake (kcal/day), Mediterranean Diet Score (MDS, continuous), and antioxidant supplement use (0/1) in order to address potential dietary confounding.

This modeling (M ~ Status (UC/CD) + Age + Sex + Smoking + BMI + Disease duration + hs-CRP + ALB + Total energy intake + MDS + Antioxidant supplement use) aimed to identify which factors independently influenced oxidative and antioxidant marker levels within each disease phenotype, beyond univariate associations.

The resulting models are summarized in Table 9 for UC and Table 10 for CD, presenting standardized coefficients (β), 95% confidence intervals, and *p*-values for each predictor, along with adjusted R^2^ values indicating model fit.

##### MLR for Each Model in the UC Group (Table 9)

8-epi-PGF_2_α model

The model explained a limited proportion of variance in serum 8-epi-PGF2α levels (R^2^ = 0.192; adjusted R^2^ = 0.009), indicating that the included demographic, inflammatory, and dietary predictors accounted for only a small part of the overall variability.

In the UC group, the revised 8-epi-PGF2α model showed that disease duration emerged as an independent positive predictor (*β* = 6.38, *p* = 0.017), indicating that longer disease duration was associated with higher lipid peroxidation after adjustment for dietary factors. Neither total energy intake, MDS, nor antioxidant supplement use significantly influenced 8-epi-PGF2α levels.

SOD1 model

This model showed limited-to-moderate explanatory power (R^2^ = 0.190; adjusted R^2^ = 0.006), suggesting that the included predictors explained only a modest fraction of the variability in SOD1. While the overall fit was slightly improved after including dietary covariates, the adjusted R^2^ remained close to zero, indicating that the predictors included in the model explained only a modest fraction of SOD1 variability. These findings suggest that serum SOD1 activity in UC may be influenced by other unmeasured determinants beyond the clinical, inflammatory, and dietary variables considered here.

GPX1 model

The GPX1 model showed the highest explanatory capacity among the UC models, although it still accounted for only a modest proportion of variance (R^2^ = 0.295; adjusted R^2^ = 0.134). This indicates that approximately 30% of the variability in GPX1 could be explained by the included predictors, with a more meaningful contribution than that observed for 8-epi-PGF2α or SOD1.

For GPX1, MDS_continuous emerged as a significant positive predictor (*β* = 144.92, *p* = 0.022), while all other variables, including energy intake and supplement use, were non-significant. This suggests that a more favorable dietary pattern may be associated with enhanced glutathione peroxidase activity in UC.

##### MLR for Each Model in the CD Group (Table 10)

8-epi-PGF2α model

The model demonstrated moderate explanatory power for serum 8-epi-PGF2α (R^2^ = 0.440; adjusted R^2^ = 0.211), indicating that the included predictors explained a substantial proportion of the variability in 8-epi-PGF2α levels. This indicates that the included predictors explained a larger proportion of the variability in lipid peroxidation in CD than in UC, although the adjusted R^2^ suggests that the explanatory power was more modest after accounting for model complexity.

In the CD group, no independent predictors of 8-epi-PGF2α were identified, even after including dietary variables. The association with hs-CRP remained only suggestive (*β* = 2.21, *p* = 0.129), while energy intake, MDS, and antioxidant supplement use were not significant.

SOD1 model

This model showed a **limited** explanatory power (R^2^ = 0.360; adjusted R^2^ = 0.098). Although the unadjusted R^2^ suggested a moderate fit, the adjusted R^2^ indicated that only a relatively small proportion of SOD1 variability was reliably explained by the predictors included in the model. This pattern suggests that serum SOD1 activity in CD remains only weakly captured by the available clinical, inflammatory, and dietary covariates.

For SOD1, smoking status no longer reached statistical significance after dietary adjustment, although it remained borderline inversely associated (*β* = −269.91, *p* = 0.082), indicating a possible negative effect of smoking on antioxidant defense.

GPX1 model

The GPX1 model demonstrated the strongest explanatory capacity among all CD models (R^2^ = 0.582; adjusted R^2^ = 0.411). This indicates that more than half of the observed variability in GPX1 was explained by the predictors included in the model, and that the model retained good explanatory performance even after adjustment for the number of covariates. These findings suggest that GPX1 is more closely linked to the combined influence of metabolic, inflammatory, and dietary factors in CD than the other oxidative stress biomarkers.

For GPX1, BMI (*β* = 18.63, *p* = 0.026) and hs-CRP (*β* = 5.04, *p* = 0.003) remained significant positive predictors, whereas MDS, energy intake, and supplement use were not associated with GPX1 levels. These findings suggest that, in CD, GPX1 activity is more closely linked to inflammatory and metabolic factors than to dietary pattern per se.

Overall, the inclusion of dietary covariates did not materially alter the main regression patterns, indicating that the observed associations between oxidative stress biomarkers and disease-related variables were not primarily driven by dietary confounding.

#### 3.3.2. Ridge Logistic Regression Analysis

Ridge logistic regression models were fitted separately for UC and CD to identify independent predictors of severe disease activity.

Model adjusted for demographic (age, sex), clinical (BMI, disease duration, smoking status), inflammatory (hs-CRP, ALB), oxidative stress biomarkers (8-epi-PGF2α, SOD1, GPX1), treatments (such as 5-ASA, steroids, immunomodulators, biologics), and dietary covariates—namely, total energy intake (kcal/day), Mediterranean Diet Score (MDS, continuous), and antioxidant supplement use (0/1)—to account for potential dietary confounding. Model performance was assessed using ROC curves and the corresponding AUC.

Table 11 and Table 12 present the standardized coefficients (*β*), odds ratios per 1 SD (OR per 1 SD), and the direction of effect for each biochemical, clinical, and pre-analytical variable included in the models.

In UC, the probability of severe disease activity is predominantly determined by a combination of clinical, inflammatory, and oxidative stress factors, while dietary variables have a limited impact.

- Clinical-therapeutic factors remain highly relevant: the use of steroids (OR ≈ 1.65) and biological therapies (OR ≈ 1.47) is associated with increased probability of severe disease, reflecting their use in patients with more advanced or refractory forms. Conversely, 5-ASA treatment (OR ≈ 0.33) is strongly protective, characteristic of patients with mild-to-moderate disease. The model appropriately captures this therapeutic gradient.
- Clinical–demographic predictors also contribute: age (OR ≈ 1.56) is positively associated with severity, suggesting that older patients are more likely to develop severe forms. This finding is consistent with the literature, which shows that longer disease evolution and greater cumulative inflammatory burden are associated with more severe phenotypes.
- Inflammatory and oxidative stress biomarkers play a central role:
  – CRP (OR ≈ 1.43) remains a robust marker of systemic inflammation and is consistently associated with increased disease severity.
  – GPX1 (OR ≈ 2.30) emerges as the strongest predictor in the model, indicating a pronounced activation of the antioxidant defense system in severe disease, likely reflecting a compensatory response to sustained oxidative stress.
  – 8-epi-PGF2α (OR ≈ 1.20) shows a modest positive association, suggesting the presence of lipid peroxidation, although its contribution to severity classification is less prominent than that of GPX1.
  – SOD1 (OR ≈ 1.12) has a weaker effect, indicating a more limited role in discriminating disease severity in UC.
- Dietary variables (energy intake, MDS, antioxidant supplements) show minimal influence on the probability of severe disease, with effect sizes close to neutrality, suggesting that the observed associations are not primarily driven by dietary confounding.

In CD, the probability of severe disease activity is predominantly driven by a strongly activated inflammatory and oxidative stress profile, while clinical and dietary variables play a more limited role.

- Inflammatory and oxidative stress biomarkers represent the core determinants of severity:
  – GPX1 (OR ≈ 2.70) is the most influential predictor, indicating a marked activation of the antioxidant defense system in severe disease, likely reflecting a sustained oxidative burden.
  – CRP (OR ≈ 2.66) shows a strong association with severity, confirming the central role of systemic inflammation in CD.
  – 8-epi-PGF2α (OR ≈ 1.74) further supports the contribution of lipid peroxidation, suggesting that oxidative damage is an integral component of the severe phenotype.
  – SOD1 (OR ≈ 1.25) shows a weaker but consistent positive association.
- Clinical–demographic factors have a comparatively smaller impact:
  – Age (OR ≈ 1.24) shows a modest positive association with severity.
  – BMI and disease duration exhibit minimal or negligible effects, suggesting that, in CD, disease severity is less dependent on baseline anthropometric or temporal factors.
- Therapeutic variables contribute moderately:
  – The use of steroids (OR ≈ 1.50) and biologic therapies (OR ≈ 1.38) is associated with increased probability of severe disease, reflecting treatment allocation to more advanced cases rather than a causal effect.
  – 5-ASA (OR ≈ 0.87) shows a weak protective association, consistent with its use in less severe phenotypes.
- Dietary variables (energy intake, MDS, antioxidant supplements) have minimal influence on severity prediction, with effect sizes close to neutrality, indicating that the strong predictive performance of the model is not primarily driven by dietary factors.

##### Diagnostic Performance of Ridge Logistic Regression Models

**Figure 4:**
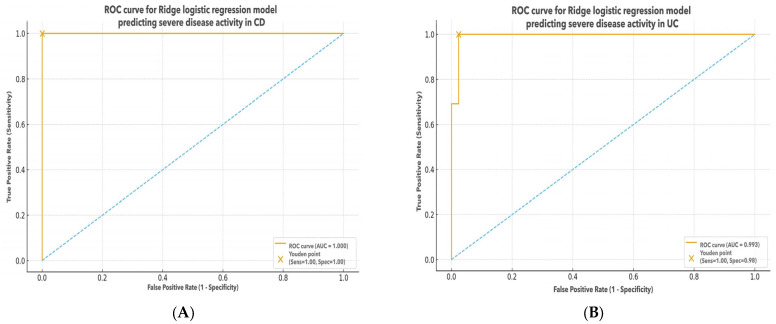
Receiver operating characteristic (ROC) curve for Ridge logistic regression model predicting severe disease activity in Crohn’s disease (CD) (**A**) and ulcerative colitis (UC) (**B**). A. The model achieved perfect classification within the sample, with an area under the curve (AUC) of 1.000, and sensitivity and specificity of 1.00 at the optimal Youden point (red marker). The ROC curve sharply rises to the upper-left corner, reflecting complete separation between severe and mild/moderate cases. B. The model demonstrated excellent discriminative performance, with an AUC of 0.993. The optimal threshold, determined using the Youden Index, achieved a sensitivity of 1.00 and a specificity of 0.98, as indicated by the red marker on the curve. The diagonal dashed line represents the reference line corresponding to no discrimination.

The ridge logistic regression model demonstrated a high diagnostic performance for identifying severe disease activity in patients with UC. The model achieved an AUC of 0.993, indicating excellent overall discriminative ability. After the inclusion of dietary covariates (total energy intake, Mediterranean Diet Score, and antioxidant supplement use), the model’s performance remained essentially unchanged, suggesting that dietary factors did not materially influence classification accuracy. At the optimal cut-off determined by the Youden Index, the model reached a sensitivity of 1.00 and a specificity of 0.98, correctly identifying all severe cases while misclassifying only a very small proportion of mild/moderate cases. The ROC curve showed a steep trajectory with minimal false-positive rates, supporting a robust separation between severe and non-severe UC phenotypes. These results confirm that the combined panel of demographic, clinical, inflammatory, oxidative stress, preanalytical, therapeutic, and dietary variables provides strong classification capability in UC (Figure 4).

The ridge logistic regression model demonstrated an apparent perfect diagnostic performance for identifying severe disease activity in patients with CD, with an AUC of 1.000. At the optimal cut-off determined by the Youden Index, both sensitivity and specificity reached 1.00, indicating perfect separation between severe and non-severe cases within the study sample. After inclusion of dietary covariates (total energy intake, Mediterranean Diet Score, and antioxidant supplement use), the model performance remained unchanged, suggesting that dietary factors did not materially influence the classification results. However, such perfect discrimination is highly likely to reflect model overfitting, particularly given the relatively small sample size and the large number of predictors included. The ROC curve showed an immediate rise toward the upper-left corner, consistent with zero false-positive and false-negative classifications in this dataset.

Therefore, although the model suggests an excellent ability to distinguish severe CD cases, these findings should be interpreted with caution, as they may not generalize to external populations. Validation in larger, independent cohorts is required to confirm the robustness and reproducibility of this predictive performance (Figure 4).

### 3.4. Correlations Between Oxidative Stress Biomarkers and Clinical Variables

**Figure 5:**
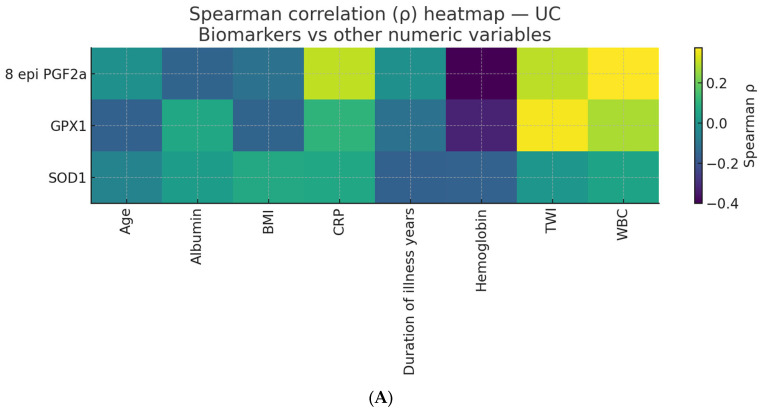

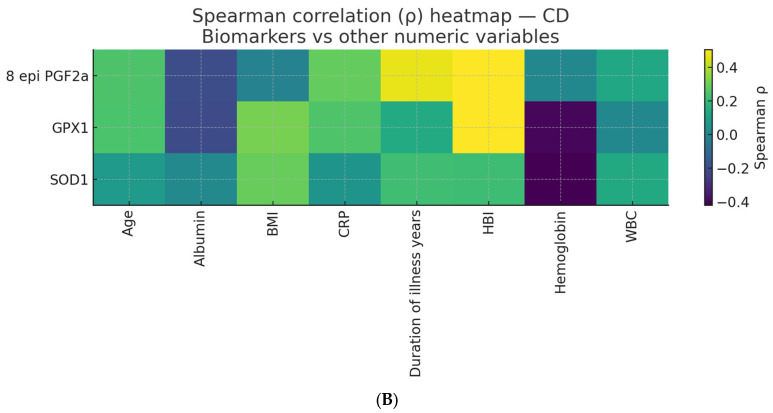
Spearman’s correlation heatmaps between oxidative stress biomarkers and clinical variables in ulcerative colitis (UC) (**A**) and Crohn’s disease (CD) (**B**). Spearman’s rank correlation coefficients (*ρ*) were calculated separately for UC and CD to assess the relationships between oxidative stress biomarkers (8-epi-PGF2α, SOD1, GPX1) and clinical or biochemical variables, including BMI, disease duration, hs-CRP, and ALB. Color intensity indicates the strength and direction of the correlation, with positive values (*p* > 0) shown in warmer tones and negative values (*p* < 0) in cooler tones. Only numeric variables were included. The correlation matrix shows interactions between oxidative stress and inflammatory activity, with stronger, more consistent correlations in UC than in CD.

Spearman’s rank correlation analysis was performed separately for UC and CD to explore associations between oxidative stress biomarkers (8-epi-PGF2α, SOD1, and GPX1) and other biochemical and clinical parameters. The correlation patterns are summarized in Table 13, including detailed correlation coefficients and *p*-values, and in Figure 5A,B (heatmaps).

In patients with UC, the correlation matrix revealed several consistent patterns linking oxidative stress biomarkers with inflammation and metabolic status (Table 13 and Figure 5A), as follows:

8-epi-PGF2α showed a strong positive correlation with hs-CRP and disease duration, supporting its function as a marker of systemic oxidative and inflammatory stress. It was inversely related to ALB, suggesting that increased oxidative lipid peroxidation is associated with poorer nutritional and protein status.

SOD1 showed moderate positive correlations with hs-CRP and GPX1, indicating coordinated activation of the antioxidant during inflammation. The correlation between SOD1 and BMI was weaker, but still suggested metabolic involvement.

GPX1 showed a positive correlation with hs-CRP and disease duration, suggesting a compensatory antioxidant response during prolonged or more active disease. The slight negative relationship between GPX1 and ALB aligns with an inflammatory–nutritional imbalance.

Overall, in UC, oxidative and antioxidant markers are closely tied to inflammatory burden (hs-CRP) and disease chronicity, indicating a strong link between redox processes and inflammation.

For CD, the correlation structure was generally weaker but showed comparable biological trends (Table 13 and Figure 5B), as follows:

8-epi-PGF2α showed a positive correlation with hs-CRP and GPX1, confirming its function as a lipid peroxidation marker in inflammatory activity. A slight inverse correlation with ALB was observed, further supporting its link to inflammatory malnutrition.

SOD1 showed positive correlations with GPX1 and hs-CRP, supporting the link between enzymatic antioxidant defenses. Its relationship with BMI and age was weak and not statistically significant.

GPX1 showed a strong correlation with hs-CRP and a moderate correlation with disease duration, indicating that a longer disease course is associated with increased oxidative response capacity.

Collectively, in CD, oxidative biomarkers correlate more closely with inflammatory markers than in UC, possibly reflecting the heterogeneous distribution of inflammation in CD.

Summary interpretation—both diseases show a consistent oxidative–inflammatory correlation profile.

- 8-epi-PGF2α functions as a pro-oxidant indicator of lipid peroxidation associated with inflammation (↑ hs-CRP, ↓ ALB);
- SOD1 and GPX1 indicate a compensatory antioxidant response that correlates with disease severity and chronicity.

The correlations are more consistent and stronger in UC, indicating greater systemic oxidative stress than in CD.

#### 3.4.1. Spearman Correlations and Benjamini–Hochberg (FDR) Correction

To explore the interrelationships between oxidative stress biomarkers and clinical or biochemical parameters, Spearman’s rank correlation analysis was performed separately for UC and CD.

Because multiple pairwise correlations were tested, the resulting *p*-values were adjusted using the Benjamini–Hochberg procedure to control the FDR. This correction was applied independently within each disease group to minimize false positives and ensure that only robust associations were interpreted as significant. Correlations with *p* ≤ 0.05 were considered statistically significant, and those with *p* ≤ 0.10 were regarded as trends toward significance.

The corrected correlation results are summarized in Table 14 and Table 15, showing the relationships between oxidative stress markers (8-epi-PGF2α, SOD1, GPX1) and clinical variables, including hs-CRP, ALB, BMI, and disease duration.

Table 14 displays the Spearman correlations between oxidative stress biomarkers (8-epi-PGF2α, SOD1, and GPX1) and clinical or biochemical parameters in UC patients, after applying the Benjamini–Hochberg FDR correction.

8-epi-PGF2α

Positive correlations with TWI (*ρ* = 0.298, *p* = 0.027, FDR = 0.109) and hs-CRP (*ρ* = 0.301, *p* = 0.025, FDR = 0.109) indicated moderate positive associations, suggesting that increased oxidative stress (lipid peroxidation) is associated with higher disease activity and systemic inflammation. WBC (*ρ* = 0.376, *p* = 0.005, FDR = 0.051) also showed a positive trend (after FDR), supporting the association between oxidative stress and immune activation.

Negative correlation with Hb (*ρ* = −0.400, *p* = 0.003, FDR = 0.053) indicates a negative trend, suggesting that increased oxidative stress may be associated with lower Hb levels, possibly pointing to anemia of inflammation.

SOD1

None of the correlations were statistically or trend-level significant after FDR correction. Small, insignificant correlations with inflammatory or clinical parameters suggest that serum SOD1 levels may vary independently of systemic inflammatory markers in UC.

GPX1

A positive correlation with TWI (*ρ* = 0.364, *p* = 0.006, FDR = 0.051, trend) suggests that higher GPX1 activity is generally associated with increased clinical disease activity, likely reflecting a compensatory antioxidant response to inflammation.

The negative correlation with Hb (*ρ* = −0.328, *p* = 0.015, FDR = 0.058, trend) indicates an inverse relationship, which suggests that higher oxidative stress and antioxidant activation may be linked to lower Hb levels.

Table 15 summarizes the Spearman correlations between oxidative stress biomarkers (8-epi-PGF2α, SOD1, and GPX1) and clinical or biochemical parameters in patients with CD, after applying the Benjamini–Hochberg FDR correction.

8-epi-PGF2α

Positive correlations with HBI (Harvey–Bradshaw Index) (*ρ* = 0.503, *p* = 0.003, FDR = 0.040) and disease duration (*ρ* = 0.473, *p* = 0.006, FDR = 0.050, trend) were the most significant findings. These results suggest that increased lipid peroxidation is associated with longer disease duration and greater clinical activity, confirming 8-epi-PGF2α as a sensitive marker of oxidative damage in chronic inflammation.

Non-significant tendencies observed: positive but non-significant correlations were noted with CRP and WBC, and a weak negative trend with ALB.

SOD1

Negative correlation with hemoglobin (*ρ* = −0.422, *p* = 0.016, FDR = 0.057, trend) indicated a moderate inverse correlation, suggesting that increased oxidative stress may contribute to lower hemoglobin levels, possibly via inflammation-related anemia.

Other correlations with BMI, HBI, and CRP were weak and not statistically significant.

GPX1

A positive correlation with HBI (*ρ* = 0.505, *p* = 0.003, FDR = 0.040) was statistically significant, indicating that higher GPX1 levels are associated with increased disease activity. This likely reflects a compensatory antioxidant response to persistent inflammation and oxidative burden.

The negative correlation with Hb (*ρ* = −0.405, *p* = 0.021, FDR = 0.102) indicated a non-significant tendency association, consistent with the pattern seen for 8-epi-PGF2α and SOD1.

#### 3.4.2. Inter-Biomarker Correlations (Spearman + FDR Correction)

Inter-biomarker correlations (8-epi-PGF2α, SOD1, GPX1) were assessed separately for UC and CD using Spearman’s rank correlation, followed by Benjamini–Hochberg FDR correction (Table 16).

In UC, the correlation between 8-epi-PGF2α and GPX1 was positive and the strongest among pairs (*ρ* = 0.677, *p* < 0.0001, FDR < 0.0001), consistent with a compensatory antioxidant response to lipid peroxidation; the remaining pairs showed weaker, non-significant associations after FDR. In CD, positive correlations were also observed among biomarker pairs, but none remained significant after FDR (8-epi-PGF2α and GPX1, moderate correlation, *ρ* = 0.488, *p* = 0.045, FDR = 0.094), likely reflecting higher biological variability and the smaller sample size.

Overall, the directionality across diseases supports a coherent redox interplay in which lipid peroxidation aligns with enzymatic antioxidant activity.

## 4. Discussion

The present study offers new insights into the oxidative stress–antioxidant balance in IBD, focusing on 8-epi-PGF2α as a marker of lipid peroxidation. While previous studies and reviews have identified oxidative stress as a key feature of IBD pathophysiology [9,20], data specifically regarding 8-epi-PGF2α remain limited, making this study one of the first to quantify and relate this isoprostane to disease activity and antioxidant enzyme responses in UC and CD.

Previous studies examining 8-iso/8-epi-PGF2α in IBD have been limited but consistently support its role as a reliable marker of lipid peroxidation. Koláček et al. (2013) showed that natural polyphenols, such as Pycnogenol, reduced oxidative stress markers, including 8-iso-PGF2α, in children with CD, confirming that this isoprostane accurately reflects oxidative imbalance and therapeutic response [38]. Wendland et al. (2001) reported increased lipid peroxidation products and decreased antioxidant micronutrient levels in CD, while Cracowski et al. (2002) demonstrated elevated urinary F2-isoprostanes, providing early in vivo evidence that 8-iso-PGF2α reflects systemic oxidative stress [39,40]. Owczarek et al. (2010) found higher 8-iso-PGF2α levels in both UC and CD, which correlated with arginine-derivative metabolites, thereby linking oxidative damage to endothelial dysfunction [41]. Di Sabatino et al. (2016) further showed that anti-tumor necrosis factor-alpha (TNF-α) treatment significantly lowered 8-iso-PGF2α levels, paralleling reductions in platelet activation and inflammation, emphasizing its dual diagnostic and mechanistic significance [42].

In our study, serum 8-epi-PGF2α was notably higher in UC than in CD, indicating more intense oxidative lipid peroxidation in colitis. This aligns with the more superficial, continuous mucosal inflammation typical of UC, which increases ROS production by infiltrating NEUs. Conversely, patchy transmural inflammation in CD may generate localized oxidative activity, which could explain the lower circulating levels of 8-epi-PGF2α. The inclusion of a control group allowed for differentiation between disease-related changes and baseline physiological variability, confirming that the observed alterations in oxidative stress and inflammatory markers are specific to IBD rather than general population variability.

The gradual increases in 8-epi-PGF2α and GPX1 with disease activity in both UC and CD support their parallel roles in the oxidative response. The fact that SOD1 remained relatively stable suggests that the initial dismutation of superoxide radicals remains consistent at the systemic level, while lipid peroxidation (8-epi-PGF2α) and glutathione-dependent detoxification (GPX1) escalate with increasing inflammatory severity.

The antioxidant enzymes SOD1 and GPX1, along with catalase, constitute the primary enzymatic defense against ROS. SOD1 converts the superoxide radical to hydrogen peroxide, which is then reduced to water by GPX1 or catalase. This trio ensures rapid detoxification of ROS and helps to maintain redox balance at both the cellular and systemic levels. Therefore, we selected SOD1 and GPX1 as key indicators of the antioxidant response in IBD, as they represent complementary steps in neutralizing oxidative intermediates and directly counteract lipid peroxidation, measured by 8-epi-PGF2α.

Oxidative imbalance likely plays a central role in epithelial barrier disruption and immune activation in IBD. Excess ROS directly oxidizes membrane phospholipids, tight-junction proteins (e.g., occludin, claudins), and cytoskeletal elements, impairing barrier integrity and increasing intestinal permeability. This “leaky” barrier facilitates the translocation of luminal antigens, microbial components, and damage-associated molecular patterns (DAMPs) into the lamina propria, where they activate resident macrophages, dendritic cells, and innate lymphoid cells. The resulting release of pro-inflammatory cytokines (TNF-α, IL-1β, IL-6) further amplifies ROS generation, creating a self-sustaining feed-forward loop of oxidative stress and inflammation. Additionally, oxidative injury impairs epithelial restitution by inhibiting stem cell proliferation and delaying mucosal healing, thereby promoting chronicity [1,16,43,44]. Similarly, Balmus et al. (2016) observed an imbalance between antioxidant enzyme activity and ROS production in IBD and highlighted the lack of quantitative studies on specific isoforms, such as SOD1 and GPX1 [45].

Recent studies have partially clarified this gap. Mrowicka et al. (2017) evaluated antioxidant enzyme gene polymorphisms and reported increased GPX1 activity and decreased SOD activity in IBD patients, suggesting a compensatory upregulation of glutathione-dependent defenses during inflammation [46]. Furthermore, Sousa et al. (2024) showed that GPX1 deficiency in macrophages contributes to ferroptotic vulnerability in CD, linking altered GPX1 expression to persistent inflammation and tissue damage [47].

In our study, both SOD1 and GPX1 showed a positive correlation with 8-epi-PGF2α, supporting their functional connection within the redox network. The stronger link between GPX1 and 8-epi-PGF2α underscores the crucial role of glutathione-dependent detoxification in mitigating lipid peroxidation. Overall, these results highlight that oxidative stress and antioxidant defenses are interconnected, actively regulated systems in IBD pathophysiology.

Importantly, 8-epi-PGF2α showed positive correlations with clinical indices (TWI and HBI) and inflammatory biomarkers (CRP, WBC), and negative correlations with Hb and ALB. This pattern indicates the combined effects of inflammation, oxidative stress, and nutritional depletion. In regression models, no single demographic or biochemical variable independently predicted 8-epi-PGF2α, suggesting it reflects cumulative oxidative stress rather than isolated systemic parameters.

The strong correlation between 8-epi-PGF2α and GPX1 (*ρ* = 0.677, *p* < 0.0001) emphasizes a coordinated redox response in which increased oxidative damage prompts the activation of antioxidant enzymes. This supports the idea that oxidative stress and antioxidant defenses are closely connected in IBD.

From a translational standpoint, 8-epi-PGF2α represents a new, clinically significant biomarker for assessing oxidative stress in IBD. Compared to other markers such as malondialdehyde (MDA) or total antioxidant capacity (TAC), F2-isoprostanes offer greater analytical stability and specificity because they are formed non-enzymatically through free-radical-mediated peroxidation of arachidonic acid [8]. Measuring them thus directly indicates lipid oxidative damage, independent of cyclooxygenase or other enzymatic pathways.

Although the ridge logistic regression model yielded an AUC of 1.000 in CD, this result almost certainly represents overfitting driven by limited sample size and class imbalance, rather than genuine perfect discriminatory power. Perfect ROC values are uncommon in biological datasets and typically indicate model optimism when applied to small, internally evaluated samples. Thus, while the findings suggest strong discriminatory potential of oxidative stress biomarkers, particularly 8-epi-PGF2α and GPX1, the performance metrics observed in CD require replication in larger and independent cohorts. Importantly, the inclusion of dietary covariates (total energy intake, MDS, and antioxidant supplement) did not modify the model performance, further suggesting that dietary factors were not responsible for the observed classification pattern.

Comparative analysis of the UC and CD models confirms fundamental pathogenic differences between the two inflammatory bowel diseases. In UC, the probability of severe activity is strongly influenced by the therapeutic profile and clinical evolution of the patients, an aspect well documented in the recent literature [48]. The roles of corticosteroids, biologic therapies and, conversely, 5-ASA treatment in delineating severe phenotypes are consistent with current epidemiological and clinical data [49,50]. In CD, our model identified a clear profile dominated by biological biomarkers, especially GPX1, CRP, and 8-epi-PGF2α, suggesting intense activation of inflammatory and redox pathways. Importantly, these patterns remained stable after adjustment for dietary factors, suggesting that the observed differences reflect intrinsic disease mechanisms rather than dietary confounding. Independent literature confirms that, in CD, oxidative stress is a central element of pathogenesis, due to transmural inflammation and persistent immune activation [51,52].

Overall, our data suggest that UC and CD present different determinants of severity: UC is predominantly influenced by the therapeutic context, age, and moderate systemic inflammation, and CD is dominated by biological and oxidative mechanisms, with a significant redox impact on severity.

The interpretation of our findings must take into account the retrospective, cross-sectional study design. Biomarkers and clinical data were obtained at a single time point; thus, the results represent severity-stratified associations within active disease rather than temporal or causal relationships. Prospective longitudinal studies are required to evaluate how oxidative biomarkers evolve during remission, relapse, and treatment response.

### 4.1. Novelty and Specific Contribution of the Present Study

Although previous research has described various alterations in oxidative stress in IBD, the present study provides several original contributions. First, this is the first investigation to quantify serum 8-epi-PGF2α concurrently with SOD1 and GPX1 in both UC and CD, thereby offering a comprehensive view of the systemic oxidative–antioxidant balance across IBD phenotypes. Second, we demonstrate a strong, disease-activity-dependent coupling between 8-epi-PGF2α and GPX1 for the first time, suggesting a coordinated oxidative–antioxidant response not previously reported. Third, unlike prior studies that evaluated single oxidative markers or unadjusted associations, our study, by integrating FDR-corrected correlation matrices, biomarker inter-relationships, and penalized regression models, revealed a previously unreported oxidative–inflammatory network. Finally, by highlighting the stability, specificity, and clinical applicability of 8-epi-PGF2α compared with traditional markers such as MDA or TAC, this study identifies 8-epi-PGF2α as a novel, robust, and clinically meaningful biomarker for monitoring redox imbalance and disease severity in IBD.

### 4.2. Clinical Implications

The current findings demonstrate the potential of 8-epi-PGF2α as a new biomarker for evaluating oxidative stress and disease activity in IBD. Since 8-epi-PGF2α is produced by non-enzymatic lipid peroxidation, it provides a stable, specific indicator of oxidative damage, independent of enzymatic prostaglandin production.

In clinical practice, measuring serum 8-epi-PGF2α can complement traditional inflammatory markers, such as CRP or hemoglobin, by capturing the redox aspect of disease activity that is often missed by conventional tests. Its correlation with GPX1 supports the idea that oxidative stress is actively balanced by enzymatic antioxidant defenses, indicating that a combined oxidative–antioxidant profile may enhance patient stratification and treatment monitoring.

Moreover, 8-epi-PGF2α could serve as a target biomarker for antioxidant or anti-inflammatory treatments, allowing clinicians to assess the redox response to therapy, especially during biologic or nutritional interventions. Including this marker in future multicenter, long-term studies could help in developing personalized treatment plans to reduce oxidative damage and prevent disease progression in UC and CD.

Future research should explore integrating dietary assessment tools, such as the MDS, FFQ, and energy intake, with established prognostic indices, including the Prognostic Nutritional Index (PNI), the Control Nutritional Status Score (CONUT), and the Glasgow Prognostic Score (GPS). Although these approaches have been studied separately, their combined evaluation in IBD remains limited, making this an area for future research. Such an integrative framework could provide a more comprehensive understanding of the interplay among nutritional status, systemic inflammation, and oxidative stress [53,54,55].

By linking dietary patterns to validated prognostic markers, future studies may improve risk stratification, identify patients at higher risk of severe disease or suboptimal therapeutic response, and support the development of personalized nutritional and therapeutic strategies. This approach could bridge the gap between the metabolic, inflammatory, and clinical dimensions of IBD, offering a more holistic model for disease monitoring and management.

### 4.3. Study Limitations

This study has several limitations that should be acknowledged. First, the sample size was relatively small (87 patients total; 55 with UC and 32 with CD), which may limit the statistical power and the generalizability of the results. Second, all participants were recruited from a single tertiary university center (Craiova, Romania) and, although patient management followed standardized national guidelines, regional or referral bias cannot be ruled out. Third, the cross-sectional design prevents establishing causal relationships between oxidative stress biomarkers and disease activity; longitudinal studies are needed to observe how these parameters vary during remission and relapse. Additionally, only serum measurements were performed; tissue-level oxidative and antioxidant activity was not evaluated, which could provide a more localized view of redox imbalance within the intestinal mucosa.

However, a key strength of this study is the choice of 8-iso/8-epi-PGF2α (F2-isoprostane) as the oxidative stress biomarker. Unlike many redox markers, which are highly affected by diet, medication, or sample handling, 8-iso-PGF2α is easy to detect, chemically stable, persists in biological fluids, and is largely unaffected by dietary factors, as its formation is regulated by endogenous antioxidant capacity [56,57,58]. This biochemical stability strengthens the validity of our results and supports the use of 8-epi-PGF2α as a reliable systemic marker of oxidative stress in IBD.

The perfect AUC observed for CD is likely inflated by overfitting due to the small sample size and should be interpreted cautiously. Validation in larger, multi-center CD populations is needed to confirm the predictive utility of these biomarkers.

Although dietary intake was retrospectively assessed using a food frequency approach and incorporated into the statistical models, residual confounding cannot be entirely excluded due to potential recall bias and the inherent limitations of self-reported dietary data. Moreover, detailed nutrient-level analyses were not performed and, therefore, subtle dietary influences on oxidative stress biomarkers may not have been fully captured.

Despite these limitations, this study provides robust preliminary evidence supporting 8-epi-PGF2α as a novel, clinically relevant oxidative stress biomarker in IBD, laying the foundation for larger, multicenter, longitudinal validation studies.

In summary, this study highlights 8-epi-PGF2α as a promising oxidative stress biomarker in IBD, with both clinical and mechanistic relevance, and supports its robustness even after accounting for potential dietary influences. Its link to inflammation, disease activity, and antioxidant defenses emphasizes its potential for redox-based patient stratification and therapy monitoring.

## 5. Conclusions

This study shows that 8-epi-PGF2α is a sensitive and clinically relevant biomarker of oxidative stress in IBD, with higher levels observed in UC than in CD and a progressive increase over time. Its strong correlation with GPX1 reflects coordinated activation of oxidative and antioxidant pathways, highlighting the role of redox imbalance in IBD pathophysiology. The inclusion of a control group further confirms that these alterations are disease-specific rather than attributable to physiological variability.

By integrating oxidative, inflammatory, and clinical parameters, our findings support the relevance of 8-epi-PGF2α as a meaningful marker for disease monitoring. These associations remained stable after adjustment for dietary factors, supporting the robustness of the results. Although the Ridge regression model showed high discriminative ability, findings in CD should be interpreted cautiously due to potential overfitting.

From a translational perspective, 8-epi-PGF2α emerges as a promising biomarker of redox imbalance, with potential utility for disease monitoring and assessment of therapeutic response. Future multicenter and longitudinal studies are needed to validate its clinical applicability.

## Figures and Tables

**Table 3:** Demographic and clinical characteristics of patients in the UC and CD groups.

Parameters	Control (*n* = 50)	UC (*n* = 55)	CD (*n* = 32)	*p* Value
Age (yrs) (mean ± SD)	45.82 ± 5.97	47.02 ± 17.48	42.40 ± 13.40	0.295
Sex ratio (male/female), *n*	21/29	22/23	22/10	0.057
Residence, (Urban/Rural), *n*	34/16	35/20	17/15	0.863
Smoking (Yes/No), *n*	24/26	21/34	20/12	0.512
Alcohol (Yes/No)	21/29	24/31	10/22	0.449
Duration of illness	-	13.0 [1.0–22.0]	7.5 [2.0–15.0]	<0.0001
BMI (kg/m^2^) [median (range)]	24.40 [21.71–35.26]	28.40 [16.72–42.29]	27.75 [21.63–36.57]	<0.0001
Laboratory parameters				
WBC (×10^3^/μL) (mean ± SD)	6.83 ± 1.47	9.34 ± 2.89	9.46 ± 2.83	<0.0001
NEU (×10^3^/μL) (mean ± SD)	4.12 ± 1.11	6.58 ± 2.12	6.13 ± 2.56	<0.0001
LYM (×10^3^/μL) [median (range)]	1.93 [0.72–3.32]	2.02 [0.98–5.32]	2.37 [1.34–4.71]	0.016
MON (×10^3^/μL) [median (range)]	0.46 [0.25–0.95]	0.39 [0.12–1.38]	0.55 [0.18–0.91]	0.002
Assessing anemia				
Hemoglobin (g/dL) [median (range)]	13.50 [9.10–17.00]	12.40 [6.62–15.10]	12.83 [10.08–17.04]	0.0001
RDW (%) (mean ± SD)	13.02 ± 0.97	15.02 ± 2.58	13.13 ± 1.45	<0.0001
MCV (fL) [median (range)]	90.70 [81.20–99.50]	90.10 [60.20–111.80]	90.74 [77.70–101.20]	0.894
MCH (pg) (mean ± SD)	31.09 ± 1.60	30.93 ± 3.10	29.37 ± 1.78	0.003
MCHC (g/dL) (mean ± SD)	33.59 ± 4.35	33.56 ± 1.44	32.64 ± 1.54	0.282
Assessing platelet modifications				
PLT (×10^3^/μL) (mean ± SD)	247.18 ± 80.52	279.75 ± 73.21	253.13 ± 48.43	0.052
PDW (%) [median (range)]	16.00 [10.40–16.90]	16.10 [11.60–17.40]	19.64 [11.80–22.67]	0.0005
MPV (fL) (mean ± SD)	10.12 ± 0.91	10.09 ± 1.52	9.38 ± 1.64	0.034
Assessing malnutrition				
Albumin (g/dL) [median (range)]	6.20 [5.10–6.84]	3.36 [2.00–5.48]	4.30 [2.79–5.89]	<0.0001
hs-CRP (mg/dL) [median (range)]	14.00 [4.00–19.50]	18.00 [6.00–96.00]	14.63 [4.00–63.00]	0.0007
ESR (mm/1st h) (mean ± SD)	16.74 ± 6.85	49.51 ± 31.03	36.41 ± 23.81	<0.0001
FIB (mg/dL) (mean ± SD)	349.73 ± 57.50	441.30 ± 123.63	402.49 ± 151.80	0.0003

BMI: body mass index; hs-CRP: high-sensitivity C-reactive protein; ESR: erythrocyte sedimentation rate; FIB: Fibrinogen; WBC: white blood cells/leukocytes; NEU: neutrophils; LYM: lymphocytes; MON: monocytes; PLT: platelets; RDW: erythrocyte distribution width; MCV: mean corpuscular volume; MCH: mean corpuscular hemoglobin; MCHC: mean corpuscular hemoglobin concentration; MPV: mean platelet volume; PDW: platelet distribution width.

**Table 4:** Comparing the oxidative stress marker values between the UC and CD groups.

Marker [Median (Range)]	Control (*n* = 50)	UC (*n* = 55)	CD (*n* = 32)	*p* Value
8-epi-PGF2α (pg/mL)	32.97 [14.27–93.04]	137.95 [57.81–440.40]	117.84 [31.28–326.33]	<0.0001
SOD1 (pg/mL)	645.02 [271.44–938.04]	1537.40 [1079.16–2560.84]	1447.50 [869.26–2308.41]	<0.0001
GPX1 (pg/mL)	119.68 [85.11–260.55]	380.57 [118.83–989.07]	239.25 [69.45–743.75]	<0.0001

8-iso-PGF2α: 8-iso-prostaglandin F2α; SOD: superoxide dismutase; GPX: glutathione peroxidase.

**Table 5:** Comparing the oxidative stress marker values in relation to the disease activity.

Marker [Median (Range)]		Mild	Moderate	Severe	Factor	F (DFn, DFd)	*p* Value
UC (*n* = 55)	8-epi-PGF2α (pg/mL)	107.88	159.21	337.54	Row factor	F (23, 29) = 1.078	0.419
		57.81–140.50	97.03–335.08	113.91–440.40	Column factor	F (2, 29) = 0.027	<0.0001 **
	SOD1 (pg/mL)	1535.20	1539.60	1580.20	Row factor	F (23, 29) = 1.020	0.474
		1142.80–2560.90	1096.30–2218.20	1079.20–1935.80	Column factor	F (2, 29) = 0.894	0.420
	GPX1 (pg/mL)	241.05	403.16	501.09	Row factor	F (23, 29) = 1.350	0.220
		118.83–539.94	119.34–713.70	127.45–989.07	Column factor	F (2, 29) = 8.573	0.001 *
CD (*n* = 32)	8-epi-PGF2α (pg/mL)	65.76	98.57	203.44	Row factor	F (12, 17) = 1.079	0.432
		58.32–127.75	31.28–266.80	117.84–326.33	Column factor	F (2, 17) = 12.34	0.001 *
	SOD1 (pg/mL)	1314.70	1434.70	1469.00	Row factor	F (12, 17) = 0.352	0.964
		1079.20–1871.40	869.26–1858.50	1434.60–2308.40	Column factor	F (2, 17) = 0.554	0.585
	GPX1 (pg/mL)	207.11	225.49	508.40	Row factor	F (12, 17) = 1.782	0.134
		136.34–373.87	69.45–422.68	226.56–743.75	Column factor	F (2, 17) = 22.13	<0.0001 **

CD: Crohn’s disease; UC: ulcerative colitis; 8-epi-PGF2α: 8-epi-prostaglandin F2α; SOD: superoxide dismutase; GPX: glutathione peroxidase; DFn: degrees of freedom numerator; DFd: degrees of freedom denominator; * *p* < 0.05: statistically significant; **: the comparisons are extremely significant (*p* < 0.0001).

**Table 6:** Post hoc pairwise comparisons (Sidak-adjusted two-way ANOVA test) of 8-epi-PGF2α and GPX1 levels according to disease activity in ulcerative colitis (UC) and Crohn’s disease (CD).

Disease	Marker	Comparison	N a	N b	Median a	Median b	*p* Value	*p*-BH Adjusted	Significant
UC	8-epi-PGF2α	mild vs. moderate	18	24	107.88	159.21	<0.0001	<0.0001	**
		mild vs. severe	18	13	107.88	337.54	<0.0001	<0.0001	**
		moderate vs. severe	24	13	159.21	337.54	0.0001	0.0001	*
CD	8-epi-PGF2α	mild vs. moderate	10	13	65.75	98.57	0.6198	0.6198	ns
		mild vs. severe	10	9	65.75	203.44	0.0007	0.0021	*
		moderate vs. severe	13	9	98.57	203.44	0.0015	0.0023	*
UC	GPX1	mild vs. moderate	18	24	241.04	403.16	0.0338	0.0508	ns
		mild vs. severe	18	13	241.04	501.09	0.0339	0.0508	ns
		moderate vs. severe	24	13	403.16	501.09	0.1815	0.1815	ns
CD	GPX1	mild vs. moderate	10	13	207.11	225.49	0.7565	0.7565	ns
		mild vs. severe	10	9	207.11	508.4	0.0011	0.0016	*
		moderate vs. severe	13	9	225.49	508.4	0.0005	0.0014	*

8-epi-PGF2α: 8-epi-prostaglandin F2α; GPX: glutathione peroxidase; *p*-BH adj: *p*-values were adjusted using the Benjamini–Hochberg false discovery rate (FDR) correction; * *p* < 0.05: statistically significant; **: the comparisons are extremely significant (*p* < 0.0001); ns: not significant.

**Table 7:** Comparing oxidative stress marker values based on disease extent (L1–L3 classification) and disease behavior (B1–B3 classification) for CD, and disease extent (E1–E3 classification) for UC.

Marker [Median (Range)]		L1 Ileal	L2 Colonic	L3 Ileo-Colonic	Factor	F (DFn, DFd)	*p*-Value
CD (*n* = 32)	8-epi-PGF2α (pg/mL)	64.36	117.84	129.56	Row factor	F (11, 18) = 0.785	0.653
		31.28–127.80	58.32–326.33	57.74–279.76	Column factor	F (2, 18) = 2.315	0.128
	SOD1 (pg/mL)	1348.95	1550.21	1449.62	Row factor	F (11, 18) = 0.756	0.676
		1079.16–1858.52	869.26–2308.41	1126.31–1845.78	Column factor	F (2, 18) = 0.786	0.470
	GPX1 (pg/mL)	229.24	371.87	288.92	Row factor	F (11, 18) = 1.233	0.334
		69.45–422.68	123.05–743.75	84.97–510.03	Column factor	F (2, 18) = 1.453	0.260
		**B1** **non-stenosing, non-penetrating**	**B2** **stenosing**	**B3** **penetrating**	**Factor**	**F (DFn, DFd)**	***p*** **-value**
CD (*n* = 32)	8-epi-PGF2α (pg/mL)	67.15	123.53	128.00	Row factor	F (12, 17) = 1.570	0.192
		48.40–266.80	31.28–203.44	67.73–326.33	Column factor	F (2, 17) = 2.096	0.154
	SOD1 (pg/mL)	1550.21	1443.18	1406.80	Row factor	F (12, 17) = 0.786	0.659
		1164.82–2308.41	1160.57–1858.52	869.26–1837.21	Column factor	F (2, 17) = 1.716	0.210
	GPX1 (pg/mL)	229.34	337.18	233.08	Row factor	F (12, 17) = 0.700	0.732
		126.54–743.75	69.45–510.03	84.97–729.16	Column factor	F (2, 17) = 0.079	0.925
		**E1** **proctitis**	**E2** **left colitis**	**E3** **extensive colitis**	**Factor**	**F (DFn, DFd)**	***p*** **-value**
UC (*n* = 55)	8-epi-PGF2α (pg/mL)	131.99	132.55	177.32	Row factor	F (24, 28) = 2.743	0.006
		65.48–325.28	57.81–440.40	64.59–414.40	Column factor	F (2, 28) = 0.027	0.463
	SOD1 (pg/mL)	1445.34	1498.89	1618.71	Row factor	F (24, 28) = 0.996	0.500
		1096.29–2218.21	1079.16–1935.76	1177.63–2560.85	Column factor	F (2, 28) = 1.946	0.162
	GPX1 (pg/mL)	241.05	380.57	397.01	Row factor	F (24, 28) = 1.010	0.486
		119.34–522.89	118.83–989.07	216.45–731.37	Column factor	F (2, 28) = 0.320	0.729

CD: Crohn’s disease; UC: ulcerative colitis; 8-epi-PGF2α: 8-epi-prostaglandin F2α; SOD: superoxide dismutase; GPX: glutathione peroxidase; DFn: degrees of freedom numerator; DFd: degrees of freedom denominator.

**Table 8:** Post hoc pairwise comparisons (Sidak-adjusted two-way ANOVA test) of 8-epi-PGF2α levels according to the extent of disease (E1–E3) in ulcerative colitis (UC).

Disease	Marker	Comparison	N a	N b	Median a	Median b	*p* Value	*p*-BH Adjusted	Significance
UC	8-epi-PGF2α	E1 vs. E2	10	25	131.99	132.55	0.715	0.715	ns
		E1 vs. E3	10	20	131.99	177.32	0.281	0.616	ns
		E2 vs. E3	20	25	177.32	132.55	0.411	0.616	ns

8-epi-PGF2α: 8-epi-prostaglandin F2α; E1: proctitis; E2: left-sided colitis; E3: extensive colitis; ns: not significant.

**Table 9:** Summary table of Multiple Linear Regression (MLR) Coefficients estimates and significance for each model in the UC group.

Group	Marker	Predictor	Coefficient *β*	95% CI	*p*	Significance
UC	8-epi- PGF2α	const	−35.719	[−546.425, 474.987]	0.891	ns
		Sex_Male	4.079	[−49.245, 57.402]	0.881	ns
		Smoking_yes	1.087	[−59.528, 61.702]	0.972	ns
		Age	−1.022	[−3.019, 0.975]	0.316	ns
		BMI	−1.831	[−6.135, 2.473]	0.404	ns
		Disease duration	6.378	[1.138, 11.618]	0.017	*
		hs-CRP	0.217	[−0.766, 1.199]	0.666	ns
		Albumin	−12.034	[−50.632, 26.565]	0.541	ns
		Energy intake	0.002	[−0.170, 0.175]	0.978	ns
		MDS	42.088	[−39.112, 123.288]	0.310	ns
		Antioxidant supplements	−14.749	[−124.986, 95.489]	0.793	ns
UC	SOD1	const	1492.142	[312.216, 2672.068]	0.013	*
		Sex_Male	100.717	[−35.306, 236.740]	0.147	ns
		Smoking_yes	97.159	[−67.992, 262.309]	0.249	ns
		Age	1.221	[−2.852, 5.294]	0.557	ns
		BMI	5.865	[−2.948, 14.677]	0.192	ns
		Disease duration	−14.436	[−29.005, 0.132]	0.052	ns
		hs-CRP	−0.748	[−4.528, 3.031]	0.698	ns
		Albumin	−3.039	[−90.967, 84.889]	0.946	ns
		Energy intake	−0.222	[−0.622, 0.178]	0.277	ns
		MDS	50.058	[−91.915, 192.031]	0.490	ns
		Antioxidant supplements	−85.385	[−301.584, 130.814]	0.439	ns
UC	GPX1	const	−0.985	[−893.750, 891.780]	0.998	ns
		Sex_Male	−14.060	[−123.100, 94.979]	0.800	ns
		Smoking_yes	24.564	[−104.057, 153.186]	0.708	ns
		Age	0.172	[−3.706, 4.051]	0.931	ns
		BMI	−4.957	[−11.776, 1.862]	0.154	ns
		Disease Duration	1.151	[−12.909, 15.211]	0.873	ns
		hs-CRP	2.810	[−1.274, 6.893]	0.177	ns
		Albumin	−37.945	[−96.060, 20.170]	0.201	ns
		Energy intake	−0.166	[−0.445, 0.113]	0.245	ns
		MDS	144.923	[21.056, 268.790]	0.022	*
		Antioxidant supplements	−70.234	[−231.909, 91.441]	0.395	ns
Model summary for 8-epi-PGF2α: R^2^ = 0.192, Adjusted R^2^ = 0.009						
Model summary for SOD1: R^2^ = 0.190, Adjusted R^2^ = 0.006						
Model summary for GPX1: R^2^ = 0.295, Adjusted R^2^ = 0.134						

The constant (const) term represents the model intercept, corresponding to the baseline log-odds of having active disease when all other predictor variables are equal to zero. Although it has no direct clinical interpretation, this parameter defines the reference probability level used to estimate individual predictor effects (odds ratios); * *p* < 0.05: statistically significant; ns: not significant.

**Table 10:** Summary table of Multiple Linear Regression (MLR) Coefficients estimates and significance for each model in the CD group.

Group	Marker	Predictor	Coefficient *β*	95% CI	*p* Value	Significance
CD	8-epi- PGF2α	const	−5.459	[−36.038, 25.120]	0.726	ns
		Sex_Male	17.517	[−26.328, 61.361]	0.434	ns
		Smoking_yes	−22.751	[−73.015, 27.512]	0.375	ns
		Age	0.033	[−2.054, 2.119]	0.976	ns
		BMI	2.582	[−6.871, 12.035]	0.592	ns
		Disease duration	−0.044	[−7.432, 7.343]	0.991	ns
		hs-CRP	2.205	[−0.641, 5.051]	0.129	ns
		Albumin	12.031	[−28.309, 52.371]	0.559	ns
		Energy intake	0.022	[−0.040, 0.083]	0.488	ns
		MDS	−15.114	[−90.019, 59.792]	0.693	ns
		Antioxidant supplements	17.642	[−94.668, 129.953]	0.758	ns
CD	SOD1	const	49.513	[−105.385, 204.411]	0.531	ns
		Sex_Male	41.626	[−237.821, 321.074]	0.770	ns
		Smoking_yes	−269.907	[−574.416, 34.601]	0.082	ns
		Age	0.809	[−9.094, 10.712]	0.873	ns
		BMI	29.120	[−17.063, 75.303]	0.217	ns
		Disease duration	3.836	[−42.485, 50.157]	0.871	ns
		hs-CRP	2.129	[−8.229, 12.487]	0.687	ns
		Albumin	−15.480	[−272.538, 241.579]	0.906	ns
		Energy intake	−0.121	[−0.516, 0.274]	0.549	ns
		MDS	160.205	[−164.185, 484.595]	0.333	ns
		Antioxidant supplements	−136.874	[−778.004, 504.257]	0.676	ns
CD	GPX1	const	4.054	[−50.483, 58.591]	0.884	ns
		Sex_Male	84.811	[−35.345, 204.966]	0.167	ns
		Smoking_yes	−112.487	[−251.705, 26.732]	0.113	ns
		Age	1.397	[−2.783, 5.577]	0.512	ns
		BMI	18.634	[2.253, 35.015]	0.026	*
		Disease duration	−10.173	[−34.637, 14.291]	0.415	ns
		hs-CRP	5.042	[1.680, 8.404]	0.003	*
		Albumin	−36.858	[−122.387, 48.672]	0.398	ns
		Energy intake	−0.030	[−0.199, 0.138]	0.725	ns
		MDS	−7.249	[−124.031, 109.532]	0.903	ns
		Antioxidant supplements	−31.575	[−258.020, 194.871]	0.785	ns
Model summary for 8-epi-PGF2α: R^2^ = 0.440, Adjusted R^2^ = 0.211						
Model summary for SOD1: R^2^ = 0.360, Adjusted R^2^ = 0.098						
Model summary for GPX1: R^2^ = 0.582, Adjusted R^2^ = 0.411						

* *p* < 0.05: statistically significant; ns: not significant.

**Table 11:** Standardized Coefficients and Odds Ratios (OR per 1 SD) from the Ridge Logistic Regression Model Predicting Severe Disease Activity in Ulcerative Colitis (UC), diet-adjusted model.

Predictor	Coefficient (*β*)	OR per 1 SD	Direction of Effect
Age	0.447	1.563	↑ risk
BMI	−0.204	0.816	↓ risk
Duration_of_illness_years	0.108	1.114	↑ risk
CRP_mg_L	0.359	1.432	↑ risk
Albumin_g_dL	−0.114	0.892	↓ risk
8_epi_PGF2α_pg_mL	0.181	1.198	↑ risk
SOD1_ pg_mL	0.110	1.117	↑ risk
GPX1_ pg_mL	0.832	2.297	↑ risk
Sex (Male/Female)_Male	0.074	1.077	↑ risk
Smoking (Yes/No)_Yes	−0.136	0.873	↓ risk
Energy_kcal_day	0.054	1.055	↑ risk
MDS_continuous	−0.085	0.918	↓ risk
Antioxidant_supplements_0_1	−0.316	0.729	↓ risk
Biologic (Yes/No)_Yes	0.384	1.468	↑ risk
Steroid (Yes/No)_Yes	0.498	1.645	↑ risk
Immunomodulator (Yes/No)_Yes	0.150	1.162	↑ risk
5ASA (Yes/No)_Yes	−1.124	0.325	↓ risk

All predictors were standardized before model fitting (mean = 0, SD = 1). Coefficients (*β*) are penalized estimates from Ridge logistic regression, representing the direction and relative magnitude of association with severe disease activity (1 = severe; 0 = mild/moderate). Odds ratios (ORs) correspond to the change in odds of severe disease per 1 SD increase in the predictor. “Increased odds” indicate a positive association, while “Decreased odds” indicate a negative association with severe activity; ↑: increased effect; ↓: decreased effect.

**Table 12:** Standardized Coefficients and Odds Ratios (OR per 1 SD) from the Ridge Logistic Regression Model Predicting Severe Disease Activity in Crohn’s Disease (CD), diet-adjusted model.

Predictor	Coefficient (*β*)	OR per 1 SD	Direction of Effect
Age	0.214	1.239	↑ risk
BMI	−0.055	0.947	↓ risk
Duration_of_illness_years	0.038	1.039	No effect
CRP_mg_L	0.979	2.661	↑ risk
Albumin_g_dL	−0.265	0.767	↓ risk
8_epi_PGF2α_pg_mL	0.554	1.741	↑ risk
SOD1_ pg_mL	0.222	1.249	↑ risk
GPX1_ pg_mL	0.993	2.700	↑ risk
Sex (Male/Female)_Male	0.279	1.322	↑ risk
Smoking (Yes/No)_Yes	0.128	1.137	↑ risk
Energy_kcal_day	0.051	1.052	↑ risk
MDS_continuous	0.073	1.075	↑ risk
Antioxidant_supplements_0_1	0.073	1.075	↑ risk
Biologic (Yes/No)_Yes	0.324	1.382	↑ risk
Steroid (Yes/No)_Yes	0.408	1.503	↑ risk
Immunomodulator (Yes/No)_Yes	0.189	1.208	↑ risk
5ASA (Yes/No)_Yes	−0.134	0.874	↓ risk

All variables were standardized before modeling. The Ridge logistic regression model (penalty = L2) estimates coefficients that are pulled toward zero to reduce overfitting while keeping all predictors. Coefficients (*β*) show the direction and relative strength of association with severe disease activity. Odds ratios (OR per 1 SD) represent the change in odds of severe activity for each 1 SD increase in a variable. “Increased odds” indicates positive associations, while “Decreased odds” indicates inverse associations with disease severity; ↑: increased effect; ↓: decreased effect.

**Table 13:** Relationships between clinical variables and oxidative stress biomarkers.

Disease	Biomarker	Variable	*n*	Spearman (rho)	*p*-Value
UC	8-epi-PGF2α	Age	55	−0.012	0.932
		BMI	55	−0.107	0.437
		Duration_of_illness _years	55	−0.011	0.938
		TWI	55	0.298	0.027 *
		hs-CRP	55	0.301	0.025 *
		Albumin	55	−0.151	0.271
		Hemoglobin	55	−0.400	0.002 *
		WBC	55	0.376	0.005 *
UC	SOD1	Age	55	−0.054	0.698
		BMI	55	0.067	0.627
		Duration_of_illness _years	55	−0.163	0.235
		TWI	55	0.008	0.957
		hs-CRP	55	0.063	0.646
		Albumin	55	0.026	0.850
		Hemoglobin	55	−0.156	0.256
		WBC	55	0.049	0.725
UC	GPX1	Age	55	−0.158	0.248
		BMI	55	−0.150	0.273
		Duration_of_illness _years	55	−0.112	0.416
		TWI	55	0.364	0.006 *
		hs-CRP	55	0.104	0.450
		Albumin	55	0.062	0.654
		Hemoglobin	55	−0.328	0.015 *
		WBC	55	0.276	0.041 *
CD	8-epi-PGF2α	Age	32	0.247	0.173
		BMI	32	−0.010	0.958
		Duration_of_illness _years	32	0.473	0.006 *
		HBI	32	0.503	0.003 *
		hs-CRP	32	0.284	0.115
		Albumin	32	−0.202	0.268
		Hemoglobin	32	0.009	0.960
		WBC	32	0.135	0.460
CD	SOD1	Age	32	0.075	0.683
		BMI	32	0.288	0.110
		Duration_of_illness _years	32	0.219	0.229
		HBI	32	0.219	0.229
		hs-CRP	32	0.056	0.761
		Albumin	32	0.016	0.930
		Hemoglobin	32	−0.422	0.016 *
		WBC	32	0.142	0.437
CD	GPX1	Age	32	0.241	0.184
		BMI	32	0.314	0.080
		Duration_of_illness _years	32	0.144	0.433
		HBI	32	0.505	0.003 *
		hs-CRP	32	0.250	0.168
		Albumin	32	−0.205	0.260
		Hemoglobin	32	−0.405	0.021 *
		WBC	32	0.009	0.960

* *p* < 0.05: statistically significant; ns: not significant.

**Table 14:** Spearman correlations and Benjamini–Hochberg (FDR) correction, calculated for UC.

Disease	Biomarker	Variable	*n*	Spearman (*ρ*)	*p*-Value	FDR_BH
UC	8-epi-PGF2α	Age	55	−0.012	0.932	0.957
		BMI	55	−0.107	0.437	0.720
		Duration_of_illness_years	55	−0.011	0.938	0.957
		TWI	55	0.298	0.027	0.109
		hs-CRP	55	0.301	0.025	0.109
		Albumin	55	−0.151	0.271	0.546
		Hemoglobin	55	−0.400	0.003	0.051 *
		WBC	55	0.376	0.005	0.053 *
UC	SOD1	Age	55	−0.054	0.698	0.870
		BMI	55	0.067	0.627	0.870
		Duration_of_illness_years	55	−0.163	0.235	0.546
		TWI	55	0.008	0.957	0.957
		hs-CRP	55	0.063	0.646	0.870
		Albumin	55	0.026	0.851	0.957
		Hemoglobin	55	−0.156	0.256	0.546
		WBC	55	0.049	0.725	0.870
UC	GPX1	Age	55	−0.158	0.249	0.546
		BMI	55	−0.150	0.273	0.546
		Duration_of_illness_years	55	−0.112	0.416	0.720
		TWI	55	0.364	0.006	0.051 *
		hs-CRP	55	0.104	0.450	0.720
		Albumin	55	0.062	0.654	0.870
		Hemoglobin	55	−0.328	0.015	0.058 *
		WBC	55	0.276	0.042	0.142

*: tended to correlate positively (trend after FDR).

**Table 15:** Spearman correlations and Benjamini–Hochberg (FDR) correction, calculated for CD.

Disease	Biomarker	Variable	*n*	Spearman (*ρ*)	*p*-Value	FDR_BH
CD	8-epi-PGF2α	Age	32	0.247	0.173	0.402
		BMI	32	−0.010	0.958	0.960
		Duration_of_illness_years	32	0.473	0.006	0.050 *
		HBI	32	0.503	0.003	0.040 *
		hs-CRP	32	0.284	0.115	0.344
		Albumin	32	−0.202	0.268	0.429
		Hemoglobin	32	0.009	0.960	0.960
		WBC	32	0.135	0.460	0.613
CD	SOD1	Age	32	0.075	0.683	0.863
		BMI	32	0.288	0.110	0.344
		Duration_of_illness_years	32	0.219	0.229	0.423
		HBI	32	0.219	0.229	0.423
		hs-CRP	32	0.056	0.761	0.913
		Albumin	32	0.016	0.930	0.960
		Hemoglobin	32	−0.422	0.016	0.057 **
		WBC	32	0.142	0.437	0.613
CD	GPX1	Age	32	0.241	0.184	0.402
		BMI	32	0.314	0.080	0.321
		Duration_of_illness_years	32	0.144	0.433	0.613
		HBI	32	0.505	0.003	0.040 *
		hs-CRP	32	0.250	0.168	0.402
		Albumin	32	−0.205	0.260	0.429
		Hemoglobin	32	−0.405	0.021	0.102
		WBC	32	0.009	0.960	0.960

* *p* < 0.05: statistically significant; **: tended to correlate positively (trend after FDR).

**Table 16:** Spearman correlations and Benjamini–Hochberg (FDR) correction between oxidative stress biomarkers (8-epi-PGF2α, SOD1, GPX1) in ulcerative colitis (UC) and Crohn’s disease (CD).

Disease	Biomarker 1	Biomarker 2	*n*	Spearman (*ρ*)	*p*-Value	FDR_BH
UC	8-epi-PGF2α	SOD1	55	0.062	0.655	0.649
UC	8-epi-PGF2α	GPX1	55	0.677	<0.0001	<0.0001 *
UC	SOD1	GPX1	32	0.393	0.026	0.069
CD	8-epi-PGF2α	SOD1	32	0.059	0.750	0.748
CD	8-epi-PGF2α	GPX1	32	0.488	0.045	0.094
CD	SOD1	GPX1	55	−0.121	0.381	0.571

*: correlate extremely significant (*p* < 0.0001).

## Data Availability

The data used to support the findings of this study are available from the corresponding author upon reasonable request.
